# Untargeted serum metabolomics analysis of *Trichinella spiralis*-infected mouse

**DOI:** 10.1371/journal.pntd.0011119

**Published:** 2023-02-21

**Authors:** Peerut Chienwichai, Tipparat Thiangtrongjit, Phornpimon Tipthara, Joel Tarning, Poom Adisakwattana, Onrapak Reamtong

**Affiliations:** 1 Princess Srisavangavadhana College of Medicine, Chulabhorn Royal Academy, Bangkok, Thailand; 2 Department of Molecular Tropical Medicine and Genetics, Faculty of Tropical Medicine, Mahidol University, Bangkok, Thailand; 3 Mahidol Oxford Tropical Medicine Research Unit, Faculty of Tropical Medicine, Mahidol University, Bangkok, Thailand; 4 Centre for Tropical Medicine and Global Health, Nuffield Department of Clinical Medicine, University of Oxford, Oxford, United Kingdom; 5 Department of Helminthology, Faculty of Tropical Medicine, Mahidol University, Bangkok, Thailand; University of Glasgow, UNITED KINGDOM

## Abstract

**Background:**

Trichinellosis, caused by a parasitic nematode of the genus *Trichinella*, is a zoonosis that affects people worldwide. After ingesting raw meat containing *Trichinella* spp. larvae, patients show signs of myalgia, headaches, and facial and periorbital edema, and severe cases may die from myocarditis and heart failure. The molecular mechanisms of trichinellosis are unclear, and the sensitivity of the diagnostic methods used for this disease are unsatisfactory. Metabolomics is an excellent tool for studying disease progression and biomarkers; however, it has never been applied to trichinellosis. We aimed to elucidate the impacts of *Trichinella* infection on the host body and identify potential biomarkers using metabolomics.

**Methodology/Principal findings:**

Mice were infected with *T*. *spiralis* larvae, and sera were collected before and 2, 4, and 8 weeks after infection. Metabolites in the sera were extracted and identified using untargeted mass spectrometry. Metabolomic data were annotated via the XCMS online platform and analyzed with Metaboanalyst version 5.0. A total of 10,221 metabolomic features were identified, and the levels of 566, 330, and 418 features were significantly changed at 2-, 4-, and 8-weeks post-infection, respectively. The altered metabolites were used for further pathway analysis and biomarker selection. A major pathway affected by *Trichinella* infection was glycerophospholipid metabolism, and glycerophospholipids comprised the main metabolite class identified. Receiver operating characteristic revealed 244 molecules with diagnostic power for trichinellosis, with phosphatidylserines (PS) being the primary lipid class. Some lipid molecules, e.g., PS (18:0/19:0)[U] and PA (O-16:0/21:0), were not present in metabolome databases of humans and mice, thus they may have been secreted by the parasites.

**Conclusions/Significance:**

Our study highlighted glycerophospholipid metabolism as the major pathway affected by trichinellosis, hence glycerophospholipid species are potential markers of trichinellosis. The findings of this study represent the initial steps in biomarker discovery that may benefit future trichinellosis diagnosis.

## Author summary

Trichinellosis is a zoonosis caused by consuming raw meat containing infective larvae of genus *Trichinella* nematodes. Trichinellosis patients can be found globally, and severe cases may die from myocarditis and heart failure. Currently, information on the molecular mechanisms of trichinellosis and precise biomarkers for the infection are scarce. To improve disease diagnosis and treatment, better understanding in these areas is required. Therefore, this study aimed to elucidate impacts of *Trichinella* infection on the host body and identify potential biomarkers with metabolomics, a holistic approach to studying global compound changes in organisms. Our study used a mouse model of trichinellosis, and sera were collected before and three timepoints after infection and explored for metabolomic data. *Trichinella* infection affected the glycerophospholipid metabolism pathway within the host body, and glycerophospholipid species were highlighted as potential biomarkers of the disease. Lipid molecules have never been proposed as markers of trichinellosis before; hence, the development of biomarkers based on findings of our study could be a novel approach to improve disease diagnosis in the future.

## Introduction

Trichinellosis or trichinosis is a zoonosis caused by infection with parasitic nematodes of the genus *Trichinella*. There are eight species of *Trichinella* worm, of which *T*. *spiralis* is the most pathogenic to humans [1]. Approximately 10,000 people are reported to suffer from trichinellosis every year in countries around the world [2], for example, Italy [3], Romania [4], Slovakia [5], Canada [6], China [7], Vietnam [8], and Ghana [9]. The clinical manifestations of trichinellosis occur within two phases: the enteric phase and parenteral phase [1–2]. A high worm burden leads to severe complications and is associated with a 0.1–3.57% mortality rate [10].

Symptoms of trichinellosis are closely related to the life cycle of the parasite. Patients are infected by consuming raw meat containing larval nematodes; the gastric juices and enzymes in the stomach digest the meat and release infective larvae, which later develop into adults in the small intestine. A new batch of larvae are spawned from adults and later pass through intestinal mucosa into blood and lymphatic vessels [1–2,11]. Hypothetically, immune responses to growing parasites result in enteric symptoms, i.e., diarrhea, vomiting, and abdominal discomfort [12,13]; whereas parenteral symptoms occur after the larvae enter the systemic circulation and invade various organs of the body at approximately 1- to 2-weeks post infection (PI). Tissue damage caused by the larvae triggers immunopathological reactions, leading to eosinophilia, myalgia, muscle pain, weakness, fever, headaches, and facial and periorbital edema [1–2,11,13]. In severe cases, patients may develop myocarditis, heart failure, or neurological complications [2,10–11,14]. Many studies have reported that the immunological responses to invading worms are key factors contributing to the symptoms of trichinellosis. However, no studies have elucidated the molecular aspects of the impacts of *T*. *spiralis* infection on the host’s body, leaving a significant gap in our knowledge of this parasitic infection.

In addition to the ambiguous disease mechanisms, the lack of methods for the precise detection of *Trichinella* infections is another problem that should be solved. The diagnosis of trichinellosis relies, at present, on patient consumption history, clinical manifestations, and the serological response of antibodies to parasite antigens [2,15]. An enzyme-linked immunosorbent assay (ELISA) to *Trichinella* antigens is the most common laboratory technique used to identify infection; however, this method can identify only half of infected patients [2]. To improve the sensitivity, researchers have investigated many techniques to detect *Trichinella* infection from various samples, for example, feces, blood, etc. Detection of parasite’s genetic material as well as antigens from fecal samples showed advantage on early detection, however, the sensitivity was declined overtime [16–20]. None of the fecal markers so far lasted long enough to facilitate detection of the disease in the later stages. Serum is a valuable sample for trichinellosis detection, and many types of serum molecules (e.g., DNA and protein) have been investigated for their suitability as potential markers of the disease. Immunological techniques, e.g., ELISA, are the most common methods used to detect *Trichinella* antigens and their counterpart antibodies. Many parasitic proteins, for example, excretory-secretory (ES) antigens [21,22], serine protease [23], elastase-1 [24], and crude somatic antigen [25], have been used to develop diagnostic tests. Some studies found >90% sensitivity and specificity, unfortunately, barriers to the success of immunological techniques still exist. These include a window of non-reactivity in the early stages and the inability to distinguish past from current infections. Thus, alternative markers that can be used to diagnose trichinellosis in the early stages and throughout the course of infection would be beneficial for patient treatment.

Alterations to metabolite levels in host biofluids are the most common evidence for parasitic infection reported in previous articles. Changes to metabolites can be used to reflect the impact of infection on hosts and are considered interesting targets for biomarker development. The identification of affected pathways and metabolite biomarkers has been the subject of extensive metabolomics studies, as this is a robust strategy that can be used to pinpoint changes to overall metabolites in biological samples [26,27]. Park *et al*. used metabolomics to identify *Falciparum-*malaria-specific biomarkers for the blood stage of the parasite. Their study found many significantly changed metabolites could be mapped to metabolic pathways of both humans and the *Plasmodium* parasite. Interestingly, four metabolites had quantities that showed a positive correlation with the level of parasitemia, namely 3-methylindole, succinylacetone, S-methyl-L-thiocitrulline, and O-arachidonoyl glycidol, and presented potential parasite-specific metabolite biomarkers [28]. Vincent *et al*. investigated the fluctuation of metabolites in serum, urine, and cerebrospinal fluid samples from trypanosomiasis patients to identify potential biomarkers that could be used to stage the disease. Metabolomics revealed that two metabolites from cerebrospinal fluid, neopterin and 5-hydroxytryptophan, showed 100% sensitivity and specificity for distinguishing early and advanced stages of the disease. Metabolites in serum have also shown high sensitivity and specificity but not as high as molecules from the cerebrospinal fluid [29]. Regarding parasitic helminths, Lagatie *et al*. characterized a potential biomarker of ascariasis, 2-methyl-pentanoyl-carnitine, in the urine of human and pigs. The accuracy of the marker increased with worm burden and apparently decreased after anthelminthic drug was administered. Interestingly, the level of 2-methyl-pentanoyl-carnitine showed a strong association with the number of eggs per gram and worm count in an animal model, indicating the possibility of using this compound as a diagnostic marker for ascariasis [30]. Another study by Lagatie *et al*. aimed to discover novel biomarkers for *Onchocerca volvulus* infection using metabolomics and lipidomics of the serum and urine of patients. Their study proposed the use of inosine and hypoxanthine as serum markers and *cis*-cinnamoylglycine as a urine marker. The sensitivities of the three metabolites were 86.2%, 74.5%, and 82.9%, respectively [31]. Osakunor *et al*. focused on changes to metabolite levels in the serum of 83 children in an area endemic for *Schistosoma haematobium*. Their study found a significant increase in the level of metabolites related to host energy metabolism and purine metabolism [32]. Although there have been plenty of metabolomic studies on parasitic infections, there have been no such studies focusing on trichinellosis, stalling the progression of biomarker development for this disease.

In our study, we aimed to elucidate the impacts of *T*. *spiralis* infection to the hosts and identify potential biomarkers using a metabolomic approach. We infected mice with *T*. *spiralis* and recorded changes to the serum metabolome over four timepoints, including before infection, and 2-, 4-, and 8-weeks PI. Metabolites were extracted from serum samples and their levels explored with a mass spectrometer. Multivariate analysis, hierarchical clustering, pathway analysis, and receiver operating characteristic curve (ROC) analysis were used to identify affected pathways and potential biomarkers from the metabolomic data. Our findings are a first step towards a better understanding of *Trichinella* infections at the molecular level and provide insights into host-parasite interactions. Moreover, the findings also pinpointed some compounds with diagnostic potential that could lead to better detection methods and less severe outcomes from the disease.

## Methods

### Ethics statement

All procedures involving animals were approved by the Faculty of Tropical Medicine Animal Care and Use Committee (FTM-ACUC), Mahidol University (Approval number: FTM-ACUC 015/2021). Guidelines for the use of animals provided by the National Research Council of Thailand (NRCT) were strictly applied to all procedures of animal experiments.

### *T*. *spiralis* life cycle maintenance and mice infection

*T*. *spiralis* laboratory strain worms were maintained in the Department of Helminthology, Faculty of Tropical Medicine, Mahidol University, Thailand. Infection of the mouse model (3 mice) was performed by gastric lavage with 100 muscle larvae. Approximately 200 microliters of blood was collected from each mouse before infection (control group), and 2-, 4-, and 8-weeks PI by submandibular bleeding. Blood samples were allowed to clot and centrifuged at 2,000 × *g* for 10 minutes at 4°C to collect the serum, which was stored at −80°C until further analysis. At 8-weeks PI, all mice were sacrificed and *T*. *spiralis* infection was confirmed by microscopic examination of skeletal muscle.

### Metabolite extraction

All serum samples were extracted for metabolite in a batch, according to study of Lu *et al*. [33]. Briefly, 20 μL of serum was added to 80 μL of cold methanol and vigorously mixed for a minute. The solution was incubated at 4°C for 20 minutes and centrifuged at 12,000 rpm for 10 minutes. Supernatant was transferred to new tube and dried using speed vacuum (Tomy Digital Biology, Tokyo, Japan). Metabolite samples were kept at −80°C until further analysis. The extraction was performed in 3 technical replications.

### Metabolite identification by mass spectrometry

Metabolomic analysis was performed using Ultra-high performance liquid chromatography (UHPLC; Agilent 1260 Quaternary pump, Agilent 1260 High Performance Autosampler and Agilent 1290 Thermostatted Column Compartment SL, Agilent Technologies, CA, USA) connected to DuoSpray ion source electrospray ionization (ESI) quadrupole time-of-flight mass spectrometer (Q-TOF-MS) (TripleTOF 5600^+^, SCIEX, US). For UHPLC separation, 0.1% formic acid in water was prepared as mobile phase A and 0.1% formic acid in acetonitrile was prepared as mobile phase B. Mobile phase A and B was mixed in 1: 1 ratio (v/v) for resuspending dried metabolite samples. The solution was transferred to a liquid chromatography (LC) vial and kept in the auto-sampler at 6°C until analysis. Five microliters of solution was loaded into the UHPLC and separated on a C18 reversed phase column (ACQUITY UPLC BEH, 2.1 × 100 mm, 1.7 μM, Waters). Chromatographic separation was performed at a flow rate of 0.3 mL/min and 40°C. Gradient elution started at 5% B and held constant for 2 min (0.0–2.0 min). The gradient ramped to 60% B in 0.5 min (2.0–2.5 min) and to 80% B in 1.5 min (2.5–4.0 min). The gradient ramped to 100% B in 8 min (4.0–12.0 min), and held constant for 5 min (12.0–17.0 min). The gradient returned to 5% B in 0.1 min (17.0–17.1 min) with 2.9 min re-equilibration (17.1–20.0 min) until the next injection. Analyst Software version 1.7 (SCIEX) was used to acquire mass ion chromatogram and mass spectra from UHPLC-Q-TOF-MS system. Metabolomic analyses were performed in both positive (+ESI) and negative (-ESI) electrospray ionization modes. Data acquisition was performed with an information-dependent acquisition mode composed of a TOF-MS scan and 10 dependent product ion scans, utilizing the high sensitivity mode with dynamic background subtraction. TOF-MS scans covered mass range of m/z 100–1,000 and MS/MS ion scans covered a mass range of m/z 50–1,000. Equal aliquots of each metabolite sample were pooled to form the quality control (QC) samples. The QC samples were injected before, during, and after sample analysis to assess the system performance.

### Metabolite annotation with XCMS platform

Metabolomic files (.wiff and.wiff.scan) were annotated with XCMS online platform Version 3.7.1 (https://xcmsonline.scripps.edu/landing_page.php?pgcontent=mainPage) [34]. “Multigroup” was chosen for analysis of metabolomic data from 0, 2, 4, and 8 weeks after *T*. *spiralis* infection. Parameters for metabolite annotation included feature extraction, alignment, annotation, and identification. For feature extraction, positive and negative polarity was chosen for each set of data. Maximal tolerated m/z deviation was selected as 15 ppm and seconds peak width was between 5 and 20. The signal/noise threshold was 6 and minimum difference in m/z was 0.01. For alignment, allowable retention time duration was 5 second, minimum fraction was 0.5, and width of overlapping m/z was 0.015. For annotation, error was 5 ppm, m/z absolute error was 0.01, and search for isotopic features and their adduct formations. For identification, 74 common adducts were considered for database search with 5 ppm tolerance for database search. METLIN database was used in the process of metabolite annotation. Metabolomic raw data were uploaded and available via Metabolomic Workbench [35] (Study ID: ST002253) (http://dx.doi.org/10.21228/M8FX4R).

### Exploratory data analysis

Abundance and types of metabolites from XCMS online were analyzed with Metaboanalyst online platform version 5.0 (https://www.metaboanalyst.ca/) [36]. Regarding exploratory data analysis, metabolomic data was analyzed with “Statistic Analysis [One factor]” module. Data was filtered with interquartile range and normalized by quantile normalization. Data was transformed with cube root transformation and scaled with range scaling. Multivariate analysis, Partial least squares-discriminant analysis (PLS-DA), was used to visualize the separation of data among infection timepoints. The model was validated using leave-one-out cross-validation (LOOCV) method, which Q^2^ was presented for assessing PLS-DA performance. Heatmap was presented to show comprehensive alteration of metabolite profiles over the course of infection, which Euclidean distance measure and Ward clustering were applied. The analysis of variance (ANOVA) was used to identify differentially changed features, the features with adjusted *p-*value less than 0.05 and fold change more than 2.

To elaborate more on chemical class of significantly changed metabolites, metabolomic data from each timepoint was analyzed with “Enrichment Analysis” module. METLIN identification number of all significantly changed metabolites were converted to Human Metabolome Database identification number (HMDB ID) for the better compatibility with the platform. Then, metabolite IDs were searched for metabolite feature and classified according to their super-class, main-class, and sub-class of chemical structure.

### Pathway analysis

To study effects of *T*. *spiralis* infection to host’s bodies, serum metabolomic data at different timepoints were analyzed with “Pathway Analysis” module of Metaboanalyst online platform version 5.0. HMDB ID of significantly different compounds was added to the bioinformatic tools and visualized with scatter plot. Hypergeometric test was selected for enrichment method and relative-betweeness centrality was selected for topology analysis. All compounds in the KEGG pathway library were used for reference metabolome of mice (*Mus musculus*). In addition, data of serum proteome from previous study of our group [37] was integrated into metabolomic data of the current study. Uniprot protein ID of proteins and HMDB ID were input into “Joint Pathway-Analysis” module of Metaboanalyst online platform version 5.0. Organism was specified to *Mus musculus* and compound list was selected for metabolomic type. “All pathways (integrated)” was selected for pathway database and hypergeometric test was used for enrichment analysis. In addition, degree centrality and combined queries were chosen for topology measure and integration method, respectively. Afterward, data of affected pathways with *p*-value less than 0.05 was retrieved from KEGG database [38]. The identified pathways and their related pathways data from the database was used to generated interconnection maps at each timepoint.

### Biomarker enrichment

Regarding biomarker enrichment, “Biomarker Analysis” module was used for analysis. Compound names as well as their abundance were selected, and data was processed as performed in exploratory data analysis. Classical univariate receiver operating characteristic (ROC) curve was chosen for analysis. Compounds with area under the curve value more than 0.8, fold change more than 2, and T-test less than 0.05 were considered as potential biomarkers of trichinellosis at different time-point. Afterward, all potential metabolites were searched through human metabolome database (The Human Metabolome Database (HMDB)– 220,945 metabolite entities: https://hmdb.ca/) [39], and mouse metabolome databases (Chemical Entities of Biological Interest (ChEBI)– 2,987 metabolite entities: https://www.ebi.ac.uk/chebi/init.do, MetaboLights– 1,195 metabolite entities: https://www.ebi.ac.uk/metabolights/index) [40,41]. Metabolites those did not match with entities in databases were encouraged as metabolites of parasites.

## Results

### Metabolite profiles of mouse serum showed changes from 2 weeks after *T*. *spiralis* infection

After infection, *T*. *spiralis* larvae embedded themselves in skeletal muscle of all mice (S1 Fig), however, mice did not show signs of the disease. The infection did not affect general characteristics, behavior, food consumption, or weight of mice, indicating asymptomatic trichinellosis. In the contrary, metabolomic analysis found changes of molecules since 2 weeks-PI.

**Fig 1:**
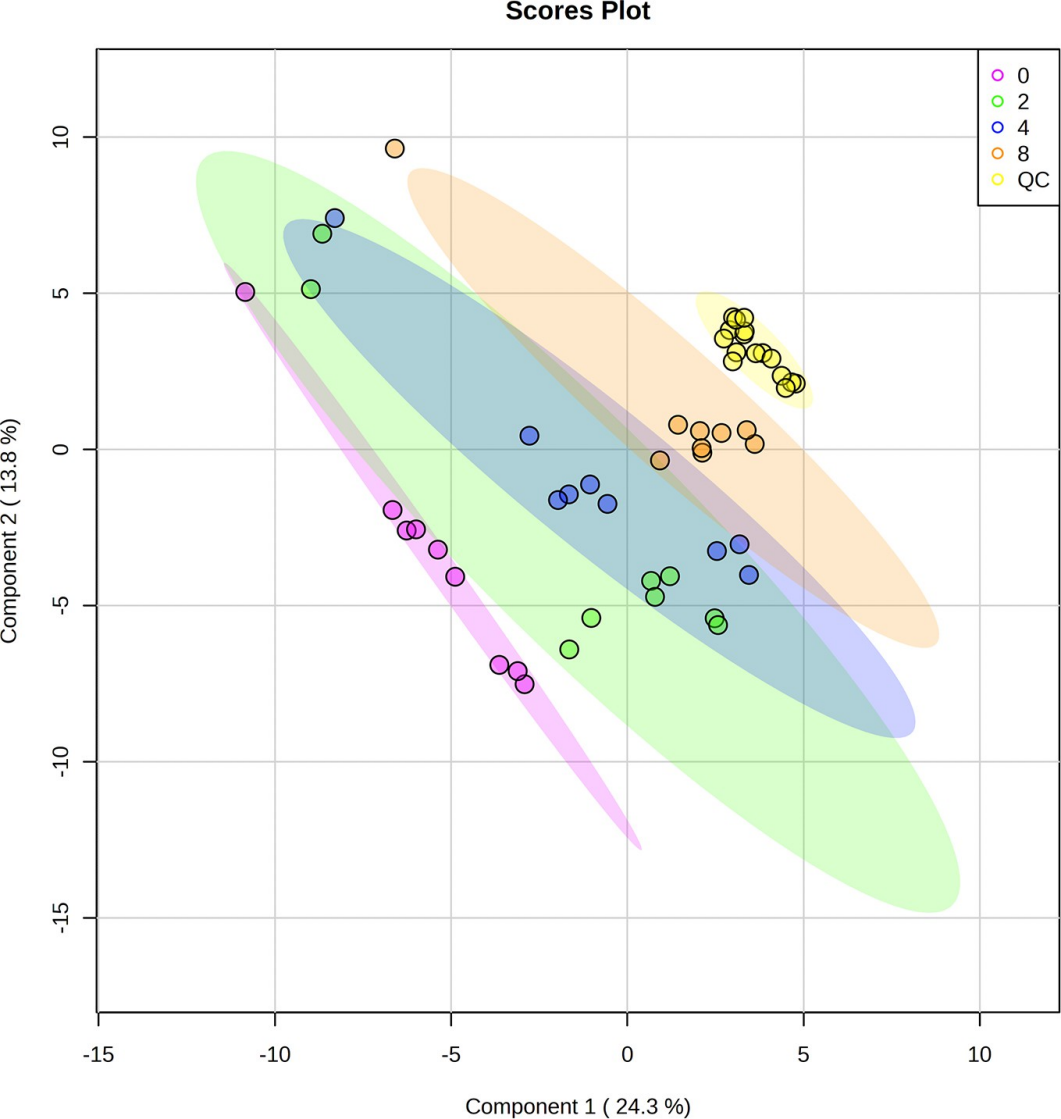
Multivariate analysis of metabolite profiles from mouse serum after *T*. *spiralis* infection, using partial least squares-discriminant analysis (PLS-DA). Violet, green, blue, and orange dots represent data from before infection, and 2-, 4-, and 8-weeks PI, respectively. QC samples were presented in yellow dots. PLS-DA showed good discrimination of metabolite profiles between different timepoints of *T*. *spiralis* infection.

**Fig 2:**
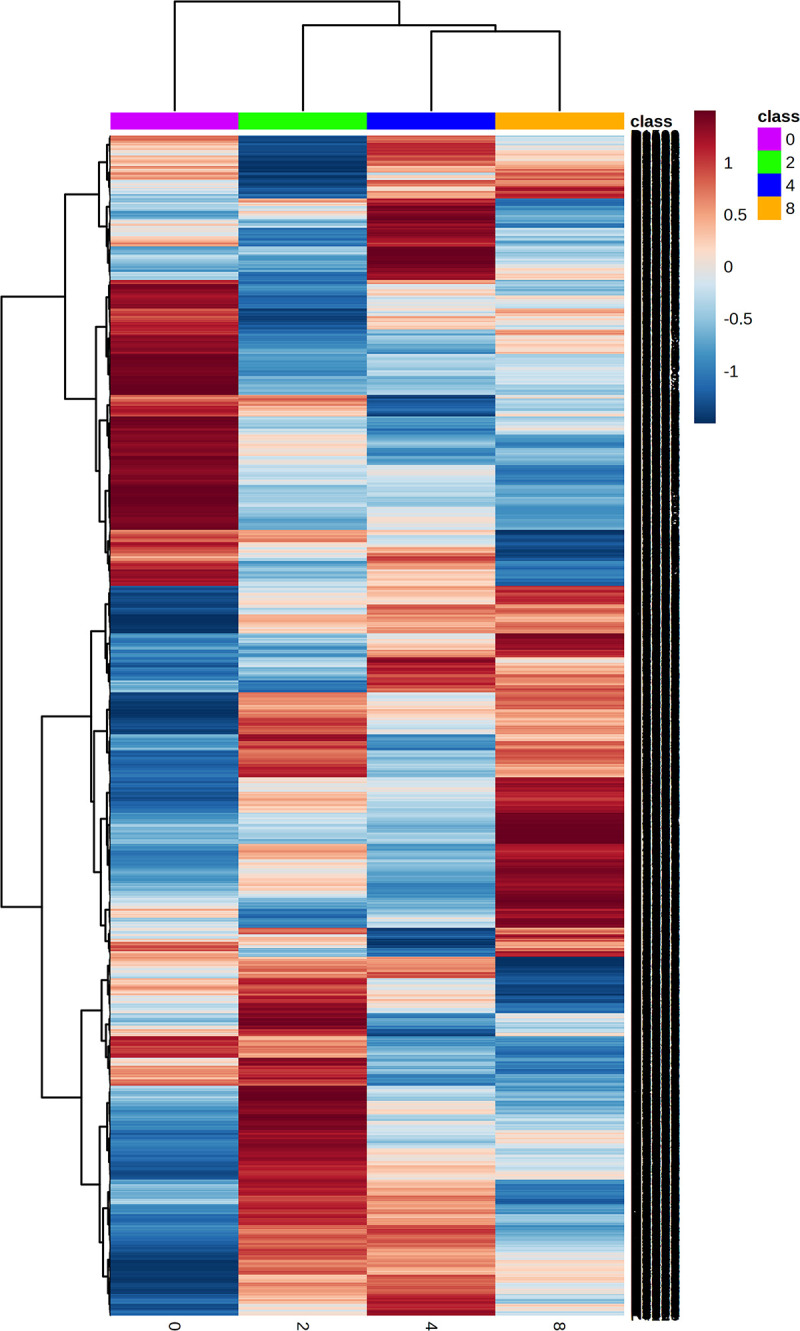
Heatmap analysis of serum metabolites after *T*. *spiralis* infection. X-axis represents experimental groups and Y-axis represents metabolite features. Red and blue color indicates higher and lower relative level, respectively. Heatmap shows dissimilar patterns of metabolite features from before infection, and 2-, 4-, and 8-weeks PI.

On analysis of the sera from the four timepoints, the mass spectrometer identified 4,688 and 5,533 metabolomic features via the positive and negative mode, respectively. Both datasets were processed and classified using supervised multivariate analysis, PLS-DA. The algorithm revealed that features from the control group (violet dots) were clustered on the left side of the plots. Features from 2-weeks PI (green dots) and 4-weeks PI (blue dots) largely overlapped in the middle of the plots, while features from 8-weeks PI (orange dots) were tightly grouped on the right-hand side (Fig 1). This finding suggested the variance of metabolites in serum of infected mice at the different time durations. In addition, QC samples were aggregated together (yellow dots), indicating the stable state of experimental conditions. The PLS-DA cross-validation showed high Q^2^ value (S2 Fig), ensuring the reliability of model for classification. Accordingly, hierarchical clustering of metabolomic data from serum samples of the 4 groups was presented with heatmap (Fig 2). The analysis showed trend of metabolite changes caused by *T*. *spiralis* infection and distinct patterns of metabolite features of 2-, 4-, and 8-weeks PI from uninfected counterparts was observed. The 2 models concordantly pointed out that *T*. *spiralis* infection had profound impact on metabolite moiety in host sera, which levels and types of affected molecules may provide some clues on pathogenesis and potential biomarkers of trichinellosis.

At 2-weeks PI, 566 features met our criteria of significantly changed metabolites in both modes of the mass spectrometer, including 41 increased and 525 decreased features. The levels of 330 features had changed after 4 weeks of infection, and these included 44 increased and 286 decreased features. At 8 weeks, 418 features showed changes in their levels following *T*. *spiralis* infection, of which 67 were increased and 351 decreased (S1 Table). The top-5 increased and decreased putative metabolites from each timepoint are shown in Table 1. Interestingly, some putative metabolites showed changes in abundance from 2-weeks PI and throughout the remainder of the experiment; for example, hesperidin (METLIN ID: 3678), malvidin 3-rutinoside (METLIN ID: 47143), and phosphoethanolamine (PE) (17:0/18:0)[U] (METLIN ID: 40464). Subsequently, we filtered our data for putative metabolites whose levels were differentially altered at all infection timepoints.

**Table 1:** Top-5 putative metabolites from each time-point after *T*. *spiralis* infection (ranked by fold change).

**No.**	**Median M/Z**	**Median Retention time (Minute)**	**Fold change**	**Adjusted *p*-value**	**Ionization mode**	**Putative metabolite**	**METLIN ID**	**Chemical formula**	**Ion adducts**	**Mass error (ppm)**
2 weeks PI										
- Increased putative metabolites										
1	202.18	1.03	15.74	2.81E^-07^	Positive	11-amino-undecanoic acid	35923	C_11_H_23_NO_2_	M+H	0
2	735.54	0.19	6.07	0.006025	Positive	N-(15Z-tetracosenoyl)-4E,6E-tetradecasphinga dienine-1-phosphoethanolamine (PE-Cer(d14: 2(4E,6E)/24:1 (15Z))	103076	C_40_H_77_N_2_O_6_P	M+Na	4
3	734.56	12.58	3.40	8.52E^-07^	Positive	1-heptadecanoyl-2-octadecanoyl-sn-glycero-3-phosphoethanolamine (PE(17:0/18:0)[U])	40464	C_40_H_80_NO_8_P	M+H	1
4	804.57	13.24	3.40	0.002712	Negative	1-octadecanoyl-2-nonadecanoyl-sn-glycero-3-phosphoserine (PS(18:0/19:0)[U])	40822	C_43_H_84_NO_10_P	M-H	2
5	577.51	18.61	2.36	0.000256	Positive	Cohibin C	90691	C_37_H_68_O_4_	M+H	1
- Decreased putative metabolites										
1	151.02	1.00	-12.64	1.69E^-09^	Negative	Xanthine	82	C_5_H_4_N_4_O_2_	M-H	1
2	633.17	7.05	-7.94	0.049853	Positive	Hesperidin	3678	C_28_H_34_O_15_	M+Na	0
3	661.17	7.05	-5.87	0.002808	Positive	Malvidin 3-rutinoside	47143	C_29_H_34_O_16_	M+Na	1
4	651.44	10.74	-5.20	0.000181	Positive	Muricoreacin	88170	C_35_H_64_O_9_	M+Na	4
5	137.04	1.03	-4.97	7.42E^-05^	Positive	Hypoxanthine	83	C_5_H_4_N_4O_	M+H	1
4 weeks PI										
- Increased putative metabolites										
1	735.54	0.19	4.04	0.006025	Positive	N-(15Z-tetracosenoyl)-4E,6E-tetradecasphinga dienine-1-phosphoethanolamine (PE-Cer(d14: 2(4E,6E)/24:1 (15Z)))	103076	C_40_H_77_N_2_O_6_P	M+Na	4
2	259.02	1.41	2.90	1.00E^-04^	Negative	D-Glucose 6-phosphate	145	C_6_H_13_O_9_P	M-H	1
3	734.56	12.58	2.89	8.52E^-07^	Positive	1-heptadecanoyl-2-octadecanoyl-sn-glycero-3-phosphoethanolamine (PE(17:0/18:0)[U])	40464	C_40_H_80_NO_8_P	M+H	1
4	804.57	13.24	2.88	0.002712	Negative	1-octadecanoyl-2-nonadecanoyl-sn-glycero-3-phosphoserine (PS(18:0/19:0)[U])	40822	C_43_H_84_NO_10_P	M-H	2
5	577.51	18.61	2.81	0.000256	Positive	Cohibin C	90691	C_37_H_68_O_4_	M+H	1
- Decreased putative metabolites										
1	113.02	0.99	-10.13	8.91E^-04^	Negative	Pteridine	5807	C_6_H_4_N_4_	M-H_2_O-H	2
2	633.17	7.05	-7.54	0.049853	Positive	Hesperidin	3678	C_28_H_34_O_15_	M+Na	0
3	330.07	1.02	-7.51	0.031704	Negative	Sanguinarine	44023	C_20_H_13_NO_4_	M-H	2
4	661.17	7.05	-4.48	0.002808	Positive	Malvidin 3-rutinoside	47143	C_29_H_34_O_16_	M+Na	1
5	559.36	14.42	-4.04	0.00268	Negative	Vitamin D3 glucosiduronate	42536	C_33_H_52_O_7_	M-H	0
8 weeks PI										
- Increased putative metabolites										
1	735.54	0.19	3.59	0.006025	Positive	N-(15Z-tetracosenoyl)-4E,6E-tetradecasphinga dienine-1-phosphoethanolamine (PE-Cer(d14: 2(4E,6E)/24:1 (15Z)))	103076	C_40_H_77_N_2_O_6_P	M+Na	4
2	366.30	8.77	2.66	0.014468	Positive	N-cis-octadec-9Z-enoyl-L-Homoserine lactone	64727	C_22_H_39_NO_3_	M+H	0
3	734.56	12.58	2.65	8.52E^-07^	Positive	1-heptadecanoyl-2-octadecanoyl-sn-glycero-3-phosphoethanolamine (PE(17:0/18:0)[U])	40464	C_40_H_80_NO_8_P	M+H	1
4	786.59	14.14	2.60	0.017015	Positive	Dioleoylphosphatidylcholine	5572	C_44_H_84_NO_8_P	M+H	1
5	786.59	10.26	2.55	0.004116	Positive	1-hexadecanoyl-2-(11E,14E-eicosadienoyl)-sn-glycero-3-phosphocholine (PC(16:0/20:2(11E, 14E))[U])	39360	C_44_H_84_NO_8_P	M+H	2
- Decreased putative metabolites										
1	151.02	1.00	-24.73	1.69E^-09^	Negative	Xanthine	82	C_5_H_4_N_4_O_2_	M-H	1
2	633.17	7.05	-8.07	0.049853	Positive	Hesperidin	3678	C_28_H_34_O_15_	M+Na	0
3	661.17	7.05	-7.25	0.002808	Positive	Malvidin 3-rutinoside	47143	C_29_H_34_O_16_	M+Na	1
4	330.07	1.02	-5.77	0.031704	Negative	Sanguinarine	44023	C_20_H_13_NO_4_	M-H	2
5	664.42	9.93	-4.87	4.38E^-05^	Negative	1-dodecanoyl-2-pentadecanoyl-glycero-3-phosphoserine (PS(12:0/15:0))	77711	C_33_H_64_NO_10_P	M-H	1

On statistical analysis, there were a total of 216 molecules whose levels were altered at all timepoints (S3 Fig). Among the compounds with a positive charge, 182 were significantly altered, e.g., 1α,25-dihydroxy-3α-methyl-3-deoxy vitamin D3 (METLIN ID: 42269), PE (19:0/16:0) (METLIN ID: 40568), and cholesta-4,6-dien-3-one (METLIN ID: 41650). In addition, 34 compounds with a negative charge were significantly altered, e.g., phosphocholine (Paz-PC) (METLIN ID: 63020) (Tables 2 and S2). To gain a better understanding of the significantly altered metabolites, the metabolites were classified according to their chemical structure.

**Table 2:** Top 15 putative metabolites from all time-points after *T*. *spiralis* infection (ranked by adjusted *p-*value those had fold change ± 2.0).

No.	Median M/Z	Median Retention time (Minute)	Fold change			Adjusted *p*-value	Ionization mode	Putative metabolite	METLIN ID	Chemical formula	Ion adducts	Mass error (ppm)
			2 weeks	4 weeks	8 weeks							
1	415.36	14.61	-3.95	-3.52	-4.00	8.52E^-07^	Positive	1α,25-dihydroxy-3α-methyl-3-deoxy vitamin D3 /1α,25-dihydroxy-3α-methyl-3-deoxycholecalciferol	42269	C_28_H_46_O_2_	M+H	0
2	734.57	12. 58	3.40	2.89	2.65	8.52E^-07^	Positive	1-nonadecanoyl-2-hexadecanoyl-sn-glycero-3-phosphoethanolamine (PE(19:0/16:0))	40568	C_40_H_80_NO_8_P	M+H	1
3	667.44	9.83	-3.17	-2.63	-3.60	6.83E^-06^	Positive	1-(9Z-tetradecenoyl)-2-(5Z,8Z,11Z,14Z-eicosatetraenoyl)-glycero-3-phosphate (PA(14:1(9Z)/20:4(5Z,8Z,11Z,14Z)))	81295	C_37_H_63_O_8_P	M+H	5
4	809.57	10.79	-3.27	-2.22	-3.50	9.79E^-06^	Positive	1-octadecyl-2-(4Z,7Z,10Z,13Z,16Z,19Z-docosahexaenoyl)-glycero-3-phospho-(1’-sn-glycerol) (PG(O-8:0/22:6(4Z,7Z,10Z, 13Z,16Z,19Z)))	79894	C_46_H_81_O_9_P	M+H	4
5	383.33	15.87	-4.01	-3.80	-2.58	1.17E^-05^	Positive	Cholesta-4,6-dien-3-one	41650	C_27_H_42_O	M+H	0
6	666.43	9.83	-4.69	-3.33	-4.58	1.70E^-05^	Positive	1-dodecanoyl-2-pentadecanoyl-glycero-3-phosphoserine (PS(12:0/15:0))	77711	C_33_H_64_NO_10_P	M+H	2
7	538.42	12.40	-2.32	-2.56	-2.13	2.34E^-05^	Positive	1-decyl-2-decyl-sn-glycero-3-phosphocholine (PC(O-10:0/O-10:0)[U])	40181	C_28_H_60_NO_6_P	M+H	1
8	788.54	13.61	-3.20	-2.23	-3.31	2.48E^-05^	Positive	1,2-di-(9Z-octadecenoyl)-sn-glycero-3-phosphoserine (PS(18:1(9Z)/18:1(9Z))[U])	40798	C_42_H_78_NO_10_P	M+H	1
9	571.43	15.87	-3.95	-3.19	-2.63	4.29E^-05^	Positive	1-tridecanoyl-2-(9Z,12Z,15Z-octadeca trienoyl)-sn-glycerol (DG(13:0/18:3(9Z, 12Z,15Z)/0:0)[iso2])	98380	C_34_H_60_O_5_	M+Na	2
10	664.42	9.93	-4.64	-3.12	-4.87	4.38E^-05^	Negative	1-O-hexadecanoyl-2-O-(9-carboxyoctano yl)-sn-glyceryl-3-phosphocholine (PAz-PC)	63020	C_33_H_64_NO_10_P	M-H	1
11	788.54	13.36	-3.55	-2.37	-3.35	5.99E^-05^	Positive	1,2-di-(9Z-octadecenoyl)-sn-glycero-3-phosphoserine (PS(18:1(9Z)/18:1(9Z)))	40815	C_42_H_78_NO_10_P	M+H	1
12	617.51	19.66	2.16	2.79	2.01	8.13E^-05^	Positive	1-(11E-octadecenoyl)-2-hexadecanoyl-sn-glycerol (DG(18:1(11E)/16:0/0:0))	4258	C_37_H_70_O_5_	M+Na	1
13	790.55	11.59	-4.44	-3.12	-3.60	8.13E^-05^	Positive	1-(9Z-octadecenoyl)-2-octadecanoyl-sn-glycero-3-phosphoserine (PS(18:1(9Z)/ 18:0)[U])		C_42_H_80_NO_10_P	M+H	1
14	399.32	13.09	-2.89	-2.48	-2.31	1.22E^-04^	Positive	(22E)-1α-hydroxy-22,23-didehydrovitamin D3 / (22E)-1α-hydroxy-22,23-didehydro cholecalciferol	42087	C_27_H_42_O_2_	M+H	1
15	772.54	14.55	-3.63	-2.29	-3.58	1.22E^-04^	Positive	1-octadecyl-2-(6Z,9Z,12Z-octadecatrieno yl)-glycero-3-phosphoserine (PS(O-18:0/ 18:3(6Z,9Z,12Z)))	78676	C_42_H_78_NO_9_P	M+H	1

### Lipids and lipid-related molecules were major metabolite classes enriched in sera of *T*. *spiralis* infected mice

**Fig 3:**
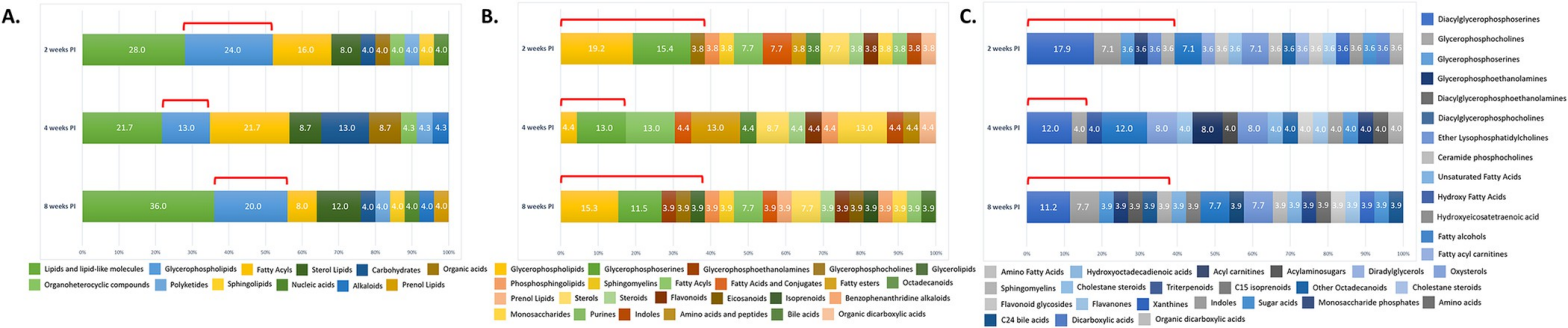
Enrichment of metabolite classes from serum of *T*. *spiralis*-infected mice at different timepoints. (A) Enrichment by super-class chemical structure. (B) Enrichment by main-class chemical structure. (C) Enrichment by sub-class chemical structure. Red brackets indicated glycerophospholipid species.

All significantly altered putative molecules from each timepoint were classified according to their super-class, main-class, and sub-class chemical structure. The distribution of the metabolites was relatively similar among chemical classes. At super-class structure, the top-3 classes of metabolites at all timepoints were lipids and lipid-like molecules, glycerophospholipids, and fatty acyls (Fig 3A and S3 Table). Accordingly, Glycerophosphoserines, Glycerophospholipids, and Fatty Acyls were the top-3 metabolite classes from main-class enrichment (Fig 3B and S3 Table). The top-3 metabolite classes from sub-class enrichment were Diacylglycerophosphoserines, Fatty alcohols, and Oxysterols (Fig 3C and S3 Table). Interestingly, lipids and lipid-related molecules was the predominant class of putative metabolites identified, especially for glycerophospholipid species. Percentage of glycerophospholipids increased with deeper structure enrichment (Fig 3, red brackets). Moreover, enrichment became significant for many molecules, for example, glycerophosphoserines, glycerophosphocholines, ether lysophosphatidylcholines. The abundances of glycerophospholipids and other lipid molecules were undoubtedly higher than that of the other classes of molecules, indicating the impact of infection on lipid moiety. We took this finding into account in our further analysis of affected pathways and biomarker identification.

### Glycerophospholipid metabolism was the major pathway altered throughout *T*. *spiralis* infection

**Fig 4:**
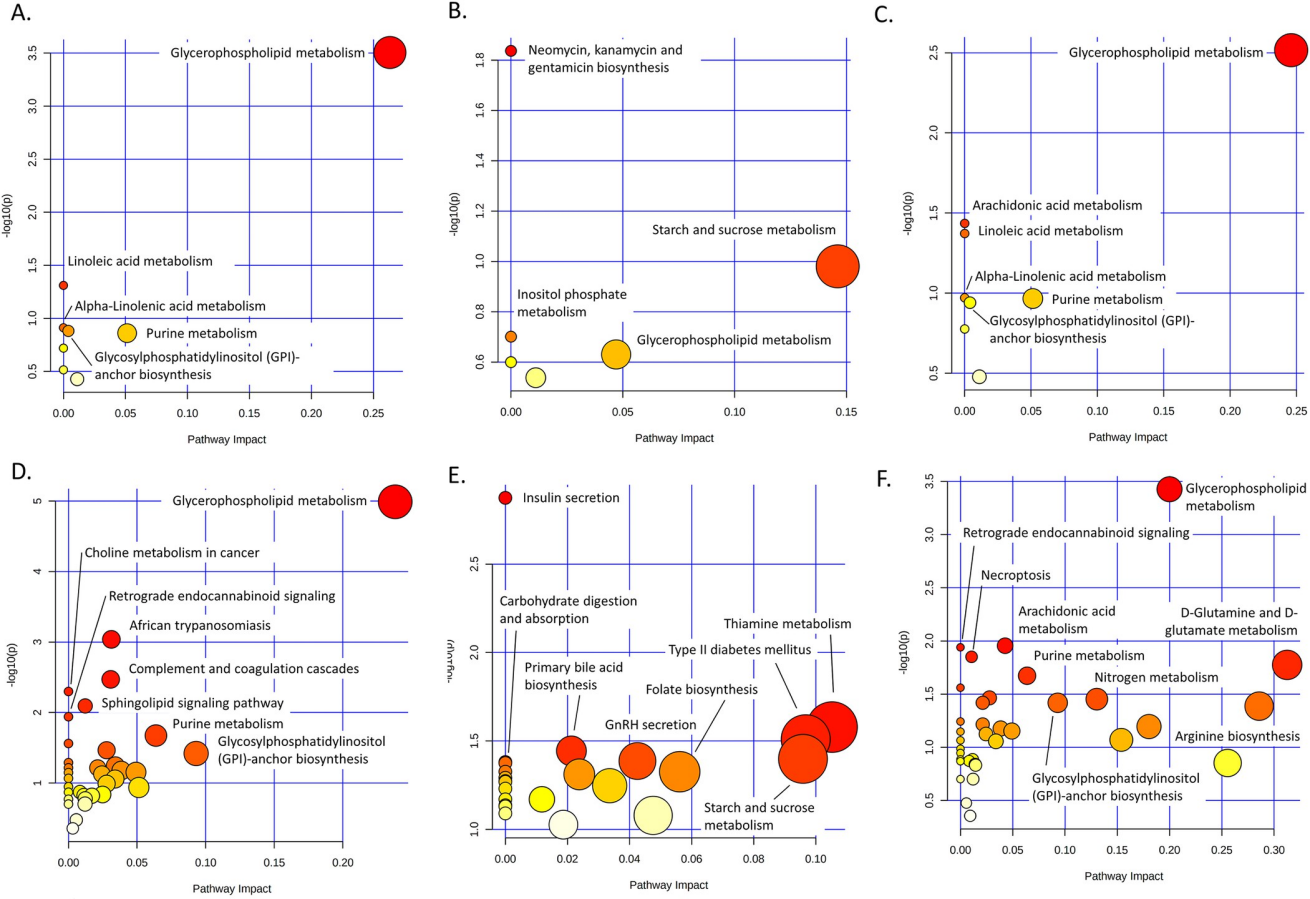
**Pathway analysis of metabolomic data and integrated proteomic and metabolomic data** (A) Metabolomic data for 2-weeks PI; (B) Metabolomic data for 4-weeks PI; (C) Metabolomic data for 8-weeks PI; (D) Combined proteomic and metabolomic data for 2-weeks PI; (E) Combined proteomic and metabolomic data for 4-weeks PI; (F) Combined proteomic and metabolomic data for 8-weeks PI. Among all identified pathways, glycerophospholipid metabolism pathway was significantly presented at 2-, and 8-weeks PI with high pathway impact. The pathway analysis highlights glycerophospholipid metabolism as the potential pathway involving with trichinellosis.

On analysis of the metabolomic data, only a few pathways were highlighted as being affected at all timepoints. At 2-weeks PI, glycerophospholipid metabolism, linoleic acid metabolism, and alpha-linoleic acid metabolism were the top-3 pathways with the lowest *p-*values (Fig 4A and S4 Table). In addition, neomycin, kanamycin, and gentamicin biosynthesis, starch and sucrose metabolism, and inositol phosphate metabolism were the top-3 pathways at 4-weeks PI (Fig 4B and S4 Table). At 8-weeks PI, the top-3 pathways were glycerophospholipid metabolism, arachidonic acid metabolism, and linoleic acid metabolism (Fig 4C and S4 Table).

To provide more comprehensive view of pathway analysis, we collected proteomic data from our previous study [37] and combined with current metabolomic data. Differentially expressed protein list of each infection duration was added to corresponding significantly altered metabolite list, then performed pathway analysis. The analysis of the combined proteomic and metabolomic data highlighted more affected pathways and with better confidence than the metabolomic data alone. At 2-weeks PI, glycerophospholipid metabolism, African trypanosomiasis, and the complement and coagulation cascades were the top-3 pathways with highest impact and *p-*values of less than 0.05 (Fig 4D and S4 Table). Thiamine metabolism, type II diabetes mellitus, and primary bile acid biosynthesis were the top-3 affected pathways in sera of mice infected for 4 weeks (Fig 4E and S4 Table). At 8-weeks PI, the top-3 affected pathways were glycerophospholipid metabolism, arachidonic acid metabolism, and retrograde endocannabinoid signaling (Fig 4F and S4 Table). Additionally, the interconnection map was generated between affected pathways and their related counterparts to visualize the connection (S4 Fig). At all timepoints, most of identified pathways were connected directly or indirectly via their related pathways. These interconnections provided overview of consequences occurred from *T*. *spiralis* infection to host’s body.

Among all affected pathways, the glycerophospholipid metabolism pathway was presented at all infection timepoints and had a high pathway impact and low *p-*value in both the metabolomic data alone and combined proteomic and metabolomic data analysis, especially at 2- and 8-weeks PI. Glycerophospholipids are a class of lipid species that are essential components of cell membranes and play vital roles in immune responses to infection. Changes in the levels of glycerophospholipids and other lipid classes may influence symptoms of disease and can be used as biomarkers for diagnosis. Therefore, we further explored the diagnostic potential of these lipid species with ROC curve analysis.

### Glycerophospholipid species, especially phosphatidylserines, were identified as potential biomarkers of trichinellosis

**Fig 5:**
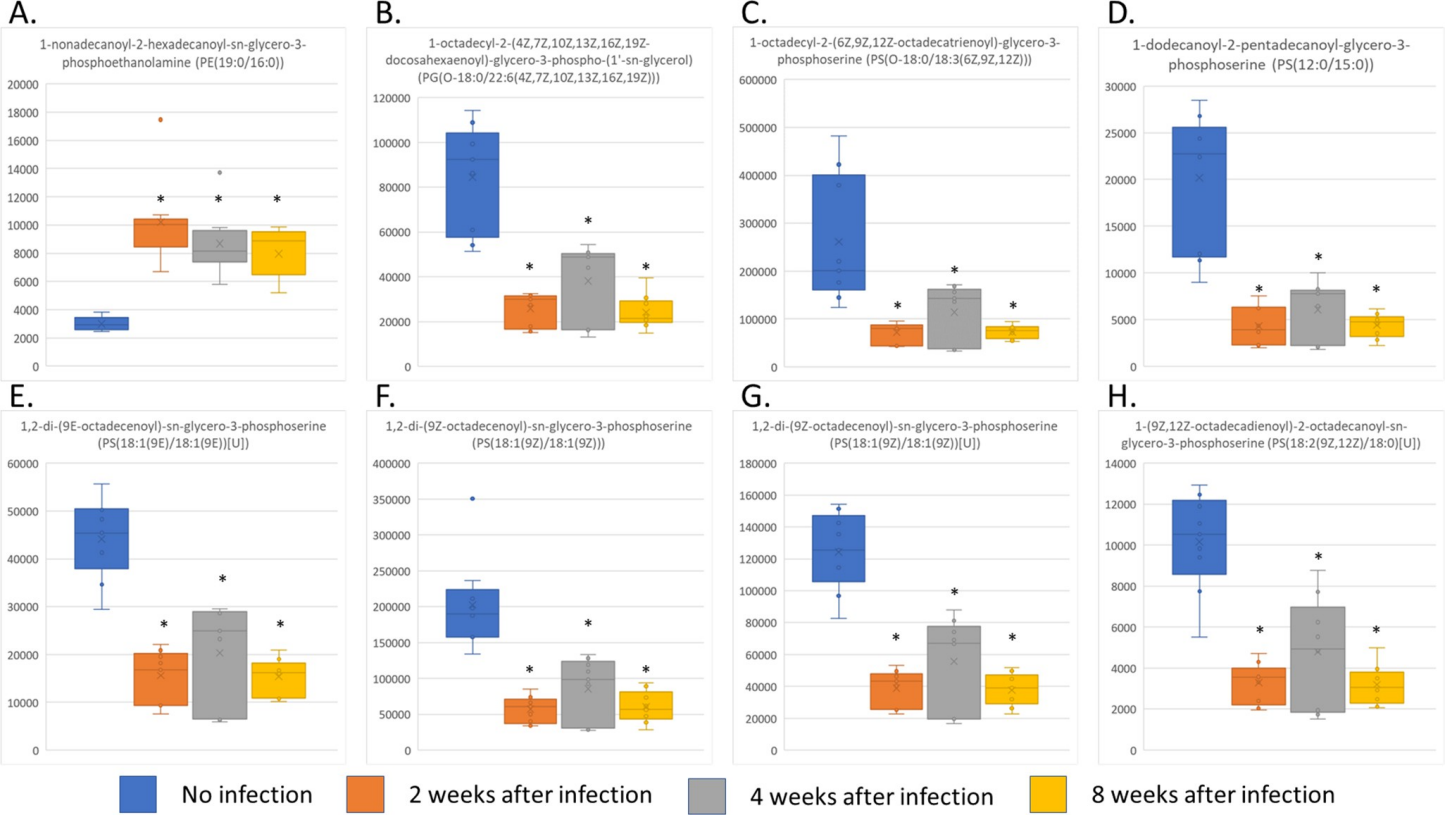
**Glycerophospholipid species that are potential biomarkers of early trichinellosis according to metabolomic analysis** (A) PE (19:0/16:0); (B) PG (O-18:0/22:6(4Z,7Z,10Z,13Z,16Z,19Z)); (C) PS (O-18:0/18:3(6Z,9Z,12Z)); (D) PS (12:0/15:0); (E) PS (18:1(9E)/18:1(9E))[U]; (F) PS (18:1(9Z)/18:1(9Z)); (G) PS(18:2(9Z,12Z)/18:0)[U]; (H) PS (18:1(9Z)/18:1(9Z)) [U]. * *p*-value < 0.01.

All altered putative metabolites were further analyzed for their biomarker potential using ROC analysis. There were 244 molecules with area under the curve (AUC) values of more than 0.8, fold changes more than 2, and T-test *p*-values of less than 0.05 (S5 Table). On integrating the metabolite classification and pathway analysis findings, 44 glycerophospholipid species were highlighted as potential biomarkers of trichinellosis. The glycerophospholipids with the highest AUC values and lowest *p*-values are shown in Fig 5, and the levels of the infected group were clearly different from those of the uninfected group. Notably, phosphatidylserine species were the main class of glycerophospholipids highlighted for biomarker selection. Thus, glycerophospholipids, especially phosphatidylserine, are an interesting class of metabolites showing potential as markers for the detection of *T*. *spiralis* infection.

To explore whether the potential phospholipid markers originated from nematodes, all highlighted metabolites were searched against publicly available human and mouse metabolome databases. From a total of 44 potential lipid markers, 10 metabolites matched entities in the databases, indicating that these compounds might have been synthesized by the hosts. Interestingly, six glycerophospholipid species were not identified in human or mouse databases and significantly increased following *T*. *spiralis* infection: phosphatidylethanolamine (PE) (19:0/16:0) (METLIN ID: 40568), Ceramide phosphoethanolamines (PE-Cer) (d14:2(4E,6E)/24:1(15Z)) (METLIN ID: 103076), PE-Cer (d14:1(4E)/22:0(2OH)) (METLIN ID: 103101), PS (18:0/19:0)[U] (METLIN ID: 40822), phosphatidic acid (PA) (O-16:0/21:0) (METLIN ID: 82161), and phosphatidylglycerol (PG) (19:0/22:2(13Z,16Z)) (METLIN ID: 79302) (S6 Table). We hypothesized that these glycerophospholipids were secreted by parasites into the host circulation. Our study strongly suggests these molecules are potential markers of trichinellosis.

## Discussion

The disease trichinellosis is one of the most neglected by the public and scientific community. More than 10,000 people are reported as having this disease every year [2], but studies devoted to this helminthic infection are markedly rare when compared with those for other parasites. The two main gaps in trichinellosis research are an understanding of the molecular impacts of the parasites on the host and the discovery of precise biomarkers for diagnosis. In our study, we used a metabolomic approach to elucidate the pathways affected by the parasites and to select potential serum biomarkers detectable 2 weeks after infection. Mice were infected with *T*. *spiralis*, and their serum samples were collected before and 2, 4, and 8 weeks after infection. Metabolomics was used to identify changes to the levels of serum molecules after infection, and bioinformatic tools were used to pinpoint molecules with biomarker potential. Our study highlighted a group of glycerophospholipids as potential markers for trichinellosis, as their levels were constantly changed since the early stage and throughout the infection.

Metabolomic analysis revealed 566, 330, and 418 significantly altered metabolites from 2-, 4-, and 8-weeks PI, respectively. Changes in the levels of metabolites are usually a consequence of endogenous and environmental factors [27], and in this case, the cause was *T*. *spiralis* infection. At 2-weeks PI, newborn larvae migrate from the small intestine to invade the host circulation and, from there, other tissues [42]. Invasion by the parasites triggers immunological reactions and inflammation in the affected organs [43]. During this period, we found reductions in the levels of two compounds involved in inflammatory processes of the host, namely xanthine (METLIN ID: 82) and hypoxanthine (METLIN ID: 83) (Table 1). Xanthine and hypoxanthine are molecules that participate in purine metabolism, i.e., the catalysis of hypoxanthine to xanthine and later to uric acid, which induces inflammation through the production of reactive oxygen species [44]. Interestingly, alterations in xanthine and hypoxanthine levels have been observed in studies of other infectious diseases [45], especially helminthic infections [46,47]. At 4-weeks PI, the enteric phase diminishes, and larvae embed themselves in striated muscle and other organs of the host, at which point patients show signs of entering the parenteral phase, e.g., muscle pain, periorbital edema, eosinophilia, etc. [42]. During this time, we found a reduction in hesperidin levels (METLIN ID: 3678) (Table 1), a compound that is related to oxidative stress and the inflammation of muscle. Hesperidin is a flavonoids class compound with anti-inflammatory properties and can induce muscle function through the stimulation of myosin-light chain phosphorylation [48]. Interestingly, a previous study showed hesperidin supplementation increased ATP production and reduced oxidative stress in muscle both *in vitro* and in mouse model [49]. Reductions in the amount of compounds with muscle-protective effects may be a consequence of helminthic invasion. In addition, decreased levels of pteridine (METLIN ID: 5807) (Table 1), a compound with fused pyrimidine and pyrazine rings [50], was observed. A derivative of pteridine, neopterin, is considered a macrophage-activation marker and is a proposed biomarker for African trypanosomiasis and leishmaniasis [29,51,52]. There is a possibility that pteridine is also involved in parasitic infection. At 8-weeks PI, all clinical manifestations usually disappear, leading to a chronic *Trichinella* infection that patients may not be aware of [42]. Alterations in the metabolites at this timepoint were similar to the changes seen at 2- and 4-weeks PI. Changes in the levels of xanthine, hesperidin, and pteridine were seen, indicating that not all impacts of infection were abolished, despite the absence of clinical symptoms.

When we considered those metabolites showing changes in their levels at all timepoints, most were glycerophospholipids. This finding correlated well with the metabolite classifications that showed glycerophospholipid species were one of the major metabolite classes. To elaborate on the pathways affected by *T*. *spiralis* infection, we performed pathway analysis with metabolomic data alone, as well as metabolomic data from this study integrated with proteomic data from a previous study by our group [37]. Changes in level of metabolites and proteins would fundamentally affect pathways and lead to impaired functions, reflecting molecular mechanism in the organism [53,54]. Integration of proteomic to metabolomic data increased the number and confidence of the identified pathways dramatically. A total of 60 biological pathways were enriched for the differentially expressed proteins and metabolites. Interestingly, some enriched pathways play regulatory roles in the progression of disease, e.g., retrograde endocannabinoid signaling and arachidonic acid metabolism (Fig 4 and S4 Table), both of which take part in inflammatory processes, a pathological consequence of larval embedding in muscle fibers [43,55,56]. During muscle invasion, host immunity attacks the parasites with many mechanisms, such as antibody production and the secretion of nitric oxides [43]. *Trichinella* larvae limit damage by modulating host immunity with their ES products. Yang *et al*. found that intraperitoneal injection of ES vesicles from *T*. *spiralis* effectively ameliorated colitis in mice. Their findings were confirmed in further histopathologic inspections and measurements of inflammatory cytokine levels [57]. Similarly, Gao *et al*. found that ES vesicles inhibited the expression of pro-inflammatory cytokines and regulated signaling pathways of inflammatory cytokines in inflamed tissue [58]. Hypothetically, ES vesicles carry numerous immunomodulatory molecules, e.g., proteins and miRNAs [57,58]. There is a possibility that EVs of *T*. *spiralis* contain anti-inflammatory compounds, as was previously reported for viral infection [59]. Noteworthy, some pathways, for examples, neomycin, kanamycin, and gentamicin biosynthesis, GnRH secretion, etc. seemed less related to trichinellosis. The interconnection maps depicted links between those pathways with others, revealed possibility that they might involve in disease progression (S4 Fig). Moreover, some metabolites or proteins in those pathways was found related with parasitic diseases. For example, D-Glucose 6-phosphate (METLIN ID: 145) in neomycin, kanamycin, and gentamicin biosynthesis pathway might associated to *Schistosoma* fluke biology [60]. These findings indicate reliability in biological significance of the pathway analysis.

Glycerophospholipid metabolism was one of the pathways enriched with high confidence (Fig 4). After combining the metabolite classification and pathway analysis findings, we hypothesized that glycerophospholipids may play some roles in trichinellosis development. Glycerophospholipids are composed of a phosphate-bound glycerol molecule and alcohol esterified with an organic molecule, such as choline or serine, and examples of glycerophospholipids include phosphatidylcholine (PC), phosphatidylethanolamine (PE), phosphatidylserine (PS), phosphatidylinositol (PI), and phosphatidylglycerol (PG). Glycerophospholipids are major constituents of cell membranes and are involved in signal transduction [61]. Nematode worms contain only 471 putative genes in lipid metabolism processes, dramatically less than those of mammals [62]. As a consequence, worms cannot *de-novo* synthesize long-chain fatty acids and sterols, which are the major building block for many lipid molecules, including glycerophospholipid. The source of the glycerophospholipids is therefore, theoretically, the host system [63]. Interestingly, Mangmee *et al*. found that glycerophospholipids comprise the major class of lipid moieties in the muscle stage of *T*. *papuae* [64]. We hypothesized that the nematodes consumed glycerophospholipid species from the circulation of the host, causing the alterations in these lipid groups observed in our metabolomic results. Alterations in the levels of molecules of glycerophospholipid metabolism pathways have been previously reported for *S*. *japonicum* infections [65,66]. These findings provide hints on the impact of helminth infection on glycerophospholipid levels in patient sera and candidates for biomarker discovery for trichinellosis. However, glycerophospholipids are complex molecules containing a variety of fatty acids, and further studies are needed to confirm the specificity of certain types of glycerophospholipids to this helminthic infection.

Metabolomics is a powerful tool for compound-based biomarker screening. In parasitic research, a number of research groups have performed metabolomic profiling to discover markers of infection. Globisch, *et al*. used metabolomics to discover biomarkers of *O*. *volvulus* infection, which they found a metabolite, N-acetyltyramine-O,β-glucuronide, that was significantly increased in urine of patients and could distinguished onchocerciasis from other helminthic infection [67,68]. Interestingly the lateral flow test developed from this metabolite had 85% accuracy, comparable to the standard antibody-based method. Addition to high accuracy, metabolite-based test can distinguish present and past infection [69]. Unfortunately, there have been no metabolomic study of attempts to find biomarkers for trichinellosis as yet. With ROC analysis, 244 features were selected as possible biomarkers, and approximately 17% of these were glycerophospholipids (S5 Table). Glycerophospholipids have been proposed as biomarkers for several diseases, including cancer [70,71], metabolic syndrome [72], asthma [73], etc. However, only a few reports have mentioned glycerophospholipids as potential markers of parasitic helminthic infection. Wewer *et al*. reported the accumulation of PE in nodular fluid on the skin of *Onchocerca ochengi*-infected cattle. They hypothesized that the nematodes secreted this glycerophospholipid species into the skin nodules, which perhaps leaked into the host circulation. However, they found very low amounts of PE in the host circulation. The PE molecules identified from nodular fluid were below the detection limit for mass spectrometry analysis of serum samples. Their study suggested that more sensitive techniques would be required for studying PE as a potential biomarker for onchocerciasis [74]. Adebayo *et al*. studied serum and urine metabolite profiles to identify biomarkers of *S*. *haematobium* infection, and they found a reduction in PC and PE levels in patients with advanced urinary pathology. They hypothesized that alterations to these molecules and their metabolism plays significant roles in the development of bladder lesions. They also proposed these metabolites could be used as biomarkers for urinary schistosomiasis [75]. In the present study, PS molecules were the most abundant glycerophospholipids identified in the ROC analysis, and almost all their abundances decreased after *T*. *spiralis* infection. Interestingly, Retra *et al*. compared a variety of glycerophospholipids present on the tegument of *S*. *mansoni* with those on hamster red blood cells to identify unique lipid species on the parasite surface. Their study found that PS were the most abundance species on the *Schistosoma* tegument. Furthermore, some PS were clearly present on the parasite membrane but rarely occurred on host cell membranes, indicating the possibility of using PS species for the diagnosis of parasitic helminth infections [76]. Notably, two PS species identified in the present study, PS (18:1(9Z)/18:2(9Z,12Z))[U] (METLIN ID: 40803) and PS (18:2(9Z,12Z)/18:0)[U] (METLIN ID: 40817), were found in the study by Retra *et al*. This coincidence may reflect the importance of PS alterations in parasitic infections.

In addition to glycerophospholipids, 44 other molecules were screened against human and mouse metabolome databases with the aim of pinpointing nematode-derived molecules present in the host circulation. We found only 10 molecules that were present in the human database, and no molecules matched with entities in the mouse databases. The human metabolome database (HMDB) contains 220,945 registered metabolites [39]. In contrast, the mouse metabolome databases, ChEBI and Metabolight, contain only 2,987 and 1,195 entities, respectively. We hypothesized that the notable difference in the numbers of registered metabolites is the main reason that no mouse database metabolites matched to our findings, especially the molecules with high diversity like glycerophospholipids. The six glycerophospholipid species that had significantly raised levels after infection and were not present in the human and mouse databases were assumed to be parasite-secreted metabolites. The six molecules have never been reported in any study of parasitic infections; therefore, we proposed these molecules may represent novel markers for *T*. *spiralis* infection.

Though our study successfully identified potential affected pathways from *Trichinella* infection and putative metabolites for biomarker development, there are some clear limitations remain. Firstly, this is a small-scale study. We used many statistical analyses to verify our findings, however, quantification of proposed metabolites in clinical samples are needed to validate diagnostic potential of markers from this study. Secondly, the differences between human and mouse biological pathways. Even though all mammals share the same *de novo* glycerophospholipid production mechanism, the Kennedy pathway [61]. The variability of glycerophospholipid species can occur due to differences in food, activity, and basic physiology between humans and mice. These issues should be taken for consideration in further biomarker development.

In conclusion, we successfully studied alterations in metabolites at three timepoints during *T*. *spiralis* infection of a mouse model. Our metabolomic findings indicated that glycerophospholipid metabolism was the major pathway affected by the infection. The ROC analysis revealed most of the highlighted compounds were of the PS class, and changes to these molecules might be used to determine the presence of infection. In addition, all glycerophospholipids identified from the ROC analysis were searched against human and mouse metabolome databases, and six metabolites were speculated to be parasite-secreted compounds and thus considered potential trichinellosis markers. Our findings provide a foundation for the development of lipid-based biomarkers for *Trichinella* infection. Following validation with large set of patient’s serum samples, the markers can used for disease detection, especially at the early infection. The development would aid trichinellosis diagnosis, which help reducing loss and death from the disease.

## Supporting information

S1 Fig *T*. *spiralis* larvae in skeletal muscle under light microscope. (A) *T*. *spiralis* larvae in skeletal muscle of infected mice at 8-weeks PI (100X). (B) *T*. *spiralis* larvae in skeletal muscle of infected mice at 8-weeks PI (400X). (C) *T*. *spiralis* larvae extracted from skeletal muscle (40X).
(TIF)

S2 Fig PLS-DA model cross-validation plot using leave-one-out cross-validation (LOOCV) method. The validation method shows cumulative values of R^2^ = 0.9975 and Q^2^ = 0.95387 for 5 components. This cross validation indicates good prediction and less likely for model overfitting.
(TIF)

S3 Fig Venn diagram showing alterations in serum metabolites after *T*. *spiralis* infection. Green, blue, and orange circles represent differential metabolites at 2-, 4-, and 8-weeks PI, respectively.
(TIF)

S4 Fig Interconnection of identified pathways from pathway analysis and their related pathways according to KEGG database. (A) Interconnection generated from integrated pathway analysis of data from 2-weeks PI. (B) Interconnection generated from integrated pathway analysis of data from 4-weeks PI. (C) Interconnection generated from integrated pathway analysis of data from 8-weeks PI. Red nodes represent identified pathways. Gray nodes represent related pathways. Only pathways with interconnections are presented. Many identified pathways are linked directly or indirectly via the same related pathways, indicating profound effects of infection to host body.
(TIF)

S1 Table Number of significantly changed metabolomic features in each timepoint. (DOCX)

S2 Table Significantly altered putative metabolites after *T*. *spiralis* infection by ANOVA analysis (Adjusted *p-*value <0.05). (CSV)

S3 Table Percentage and enrichment *p-*value of significantly changed metabolites in each timepoint. (DOCX)

S4 Table List of pathways those were affected by *T*. *spiralis* infection. (XLSX)

S5 Table List of potential biomarkers of trichinellosis from metabolomic analysis. (XLSX)

S6 Table Potential glycerophospholipid markers those were not identified from human and mouse databases. (DOCX)

## References

[1] Gottstein B, Pozio E, Nöckler K. Epidemiology, diagnosis, treatment, and control of trichinellosis. Clin Microbiol Rev. 2009;22(1):127–145. doi: 10.1128/CMR.00026-08 19136437 PMC2620635

[2] Shimoni Z, Froom P. Uncertainties in diagnosis, treatment and prevention of trichinellosis. Expert Rev Anti Infect Ther. 2015;13(10):1279–1288. doi: 10.1586/14787210.2015.1075394 26243167

[3] Turiac IA, Cappelli MG, Olivieri R, Angelillis R, Martinelli D, Prato R, et al. Trichinellosis outbreak due to wild boar meat consumption in southern Italy. Parasit Vectors. 2017;10(1):107. Published 2017 Feb 28. doi: 10.1186/s13071-017-2052-5 28241860 PMC5330091

[4] Neghina R, Neghina AM, Marincu I, Iacobiciu I. Trichinellosis in children and adults: a 10-year comparative study in Western Romania. Pediatr Infect Dis J. 2011;30(5):392–395. doi: 10.1097/INF.0b013e31820415ad 21099443

[5] Antolová D, Fecková M, Valentová D, Hurníková Z, Miklisová D, Avdičová M, et al. Trichinellosis in Slovakia—epidemiological situation in humans and animals (2009–2018). Ann Agric Environ Med. 2020;27(3):361–367. doi: 10.26444/aaem/125194 32955215

[6] Appleyard GD, Gajadhar AA. A review of trichinellosis in people and wildlife in Canada. Can J Public Health. 2000;91(4):293–297. doi: 10.1007/BF03404292 10986789 PMC6980012

[7] Zhang XZ, Wang ZQ, Cui J. Epidemiology of trichinellosis in the People’s Republic of China during 2009–2020. Acta Trop. 2022;229:106388. doi: 10.1016/j.actatropica.2022.106388 35231417

[8] Van De N, Thi Nga V, Dorny P, Vu Trung N, Ngoc Minh P, Trung Dung D, et al. Trichinellosis in Vietnam. Am J Trop Med Hyg. 2015;92(6):1265–1270. doi: 10.4269/ajtmh.14-0570 25846295 PMC4458836

[9] Addo HO, Majekodunmi AO, Sampane-Donkor E, Ofosu-Appiah LH, Opare D, Owusu-Okyere G, et al. Seroprevalence of *Taenia solium* and *Trichinella spiralis* among Humans and Pigs in Ghana. Biomed Res Int. 2021;2021:1031965. Published 2021 Oct 8. doi: 10.1155/2021/1031965 34660777 PMC8519675

[10] Murrell KD, Pozio E. Worldwide occurrence and impact of human trichinellosis, 1986–2009. Emerg Infect Dis. 2011;17(12):2194–2202. doi: 10.3201/eid1712.110896 22172230 PMC3311199

[11] Capó V, Despommier DD. Clinical aspects of infection with Trichinella spp. Clin Microbiol Rev. 1996;9(1):47–54. doi: 10.1128/CMR.9.1.47 8665476 PMC172881

[12] Khan WI. Physiological changes in the gastrointestinal tract and host protective immunity: learning from the mouse-Trichinella spiralis model. Parasitology. 2008;135(6):671–682. doi: 10.1017/S0031182008004381 18501042

[13] Bruschi F, Murrell KD. New aspects of human trichinellosis: the impact of new Trichinella species. Postgrad Med J. 2002;78(915):15–22. doi: 10.1136/pmj.78.915.15 11796866 PMC1742236

[14] Bruschi F, Brunetti E, Pozio E. Neurotrichinellosis. Handb Clin Neurol. 2013;114:243–249. doi: 10.1016/B978-0-444-53490-3.00019-4 23829915

[15] Costantino SN, Malmassari SL, Dalla Fontana ML, Diamante MA, Venturiello SM. Diagnosis of human trichinellosis: pitfalls in the use of a unique immunoserological technique. Parasite. 2001;8(2 Suppl):S144–S146. doi: 10.1051/parasite/200108s2144 11484340

[16] Liu XL, Ren HN, Shi YL, Hu CX, Song YY, Duan JY, et al. Early detection of Trichinella spiralis DNA in the feces of experimentally infected mice by using PCR. Acta Trop. 2017;166:351–355. doi: 10.1016/j.actatropica.2016.10.021 27983972

[17] Kong Q, Zhuo X, Yang X, Ding H, Ding J, Lou D, et al. Early Detection of Trichinella spiralis DNA in Rat Feces Based on Tracing Phosphate Ions Generated During Loop-Mediated Isothermal Amplification. J Parasitol. 2021;107(2):141–146. doi: 10.1645/19-137 33662114

[18] De-La-Rosa JL, Moran-Tlatelpa E, Medina Y, Gomez-Priego A, Correa D. Detection of circulating and fecal Trichinella spiralis antigens during experimental infection using monoclonal antibodies against the new born larvae. Parasite. 2001;8(2 Suppl):S123–S125. doi: 10.1051/parasite/200108s2123 11484334

[19] Boulos LM, Ibrahim IR, Negm AY, Aly SM. Detection of coproantigen in early trichinellosis. Parasite. 2001;8(2 Suppl):S136–S139. doi: 10.1051/parasite/200108s2136 11484337

[20] Zumaquero-Ríos JL, García-Juarez J, de-la-Rosa-Arana JL, Marcet R, Sarracent-Pérez J. Trichinella spiralis: monoclonal antibody against the muscular larvae for the detection of circulating and fecal antigens in experimentally infected rats. Exp Parasitol. 2012;132(4):444–449. doi: 10.1016/j.exppara.2012.09.016 23026455

[21] Sun GG, Wang ZQ, Liu CY, Jiang P, Liu RD, Wen H, et al. Early serodiagnosis of trichinellosis by ELISA using excretory-secretory antigens of *Trichinella spiralis* adult worms. Parasit Vectors. 2015;8:484. doi: 10.1186/s13071-015-1094-9 26394626 PMC4579640

[22] Kahsay R, Gómez-Morales MA, Rivera HN, McAuliffe I, Pozio E, Handali S. A Bead-Based Assay for the Detection of Antibodies against *Trichinella* spp. Infection in Humans. Am J Trop Med Hyg. 2021;104(5):1858–1862. Published 2021 Mar 29. doi: 10.4269/ajtmh.20-1569 33782208 PMC8103447

[23] Sun GG, Song YY, Jiang P, Ren HN, Yan SW, Han Y, et al. Characterization of a Trichinella spiralis putative serine protease. Study of its potential as sero-diagnostic tool. PLoS Negl Trop Dis. 2018;12(5):e0006485. doi: 10.1371/journal.pntd.0006485 29758030 PMC5967804

[24] Hu CX, Jiang P, Yue X, Zeng J, Zhang XZ, Song YY, et al. Molecular characterization of a *Trichinella spiralis* elastase-1 and its potential as a diagnostic antigen for trichinellosis. Parasit Vectors. 2020;13(1):97. doi: 10.1186/s13071-020-3981-y 32093735 PMC7041205

[25] Thanchomnang T, Sadaow L, Sanpool O, Intapan PM, Rodpai R, Boonroumkaew P, et al. Development of an immunochromatographic point-of-care test for detection of IgG antibody in serodiagnosis of human trichinellosis. Int J Infect Dis. 2021;111:148–153. doi: 10.1016/j.ijid.2021.08.056 34461253

[26] Wang Y, Li JV, Saric J, Keiser J, Wu J, Utzinger J, et al. Advances in metabolic profiling of experimental nematode and trematode infections. Adv Parasitol. 2010;73:373–404. doi: 10.1016/S0065-308X(10)73012-8 20627148

[27] Fernández-García M, Rojo D, Rey-Stolle F, García A, Barbas C. Metabolomic-Based Methods in Diagnosis and Monitoring Infection Progression. Exp Suppl. 2018;109:283–315. doi: 10.1007/978-3-319-74932-7_7 30535603 PMC7124096

[28] Park YH, Shi YP, Liang B, Medriano CA, Jeon YH, Torres E, et al. High-resolution metabolomics to discover potential parasite-specific biomarkers in a Plasmodium falciparum erythrocytic stage culture system. Malar J. 2015;14:122. doi: 10.1186/s12936-015-0651-1 25889340 PMC4377044

[29] Vincent IM, Daly R, Courtioux B, Cattanach AM, Biéler S, Ndung’u JM, et al. Metabolomics Identifies Multiple Candidate Biomarkers to Diagnose and Stage Human African Trypanosomiasis. PLoS Negl Trop Dis. 2016;10(12):e0005140. doi: 10.1371/journal.pntd.0005140 27941966 PMC5152828

[30] Lagatie O, Verheyen A, Van Asten S, Odiere MR, Djuardi Y, Levecke B, et al. 2-Methyl-pentanoyl-carnitine (2-MPC): a urine biomarker for patent Ascaris lumbricoides infection. Sci Rep. 2020;10(1):15780. doi: 10.1038/s41598-020-72804-y 32978457 PMC7519643

[31] Lagatie O, Njumbe Ediage E, Van Roosbroeck D, Van Asten S, Verheyen A, Batsa Debrah L, et al. Multimodal biomarker discovery for active *Onchocerca volvulus* infection. PLoS Negl Trop Dis. 2021;15(11):e0009999. doi: 10.1371/journal.pntd.0009999 34843471 PMC8659328

[32] Osakunor DNM, Mduluza T, Osei-Hyiaman D, Burgess K, Woolhouse MEJ, Mutapi F. Schistosoma haematobium infection is associated with alterations in energy and purine-related metabolism in preschool-aged children. PLoS Negl Trop Dis. 2020;14(12):e0008866. doi: 10.1371/journal.pntd.0008866 33315875 PMC7735607

[33] Lu K, Abo RP, Schlieper KA, Graffam ME, Levine S, Wishnok JS, et al. Arsenic exposure perturbs the gut microbiome and its metabolic profile in mice: an integrated metagenomics and metabolomics analysis. Environ Health Perspect. 2014;122(3):284–291. doi: 10.1289/ehp.1307429 24413286 PMC3948040

[34] Gowda H, Ivanisevic J, Johnson CH, Kurczy ME, Benton HP, Rinehart D, et al. Interactive XCMS Online: simplifying advanced metabolomic data processing and subsequent statistical analyses. Anal Chem. 2014;86(14):6931–6939. doi: 10.1021/ac500734c 24934772 PMC4215863

[35] Sud M, Fahy E, Cotter D, Azam K, Vadivelu I, Burant C, et al. Metabolomics Workbench: An international repository for metabolomics data and metadata, metabolite standards, protocols, tutorials and training, and analysis tools. Nucleic Acids Res. 2016;44(D1):D463–D470. doi: 10.1093/nar/gkv1042 26467476 PMC4702780

[36] Pang Z, Chong J, Zhou G, de Lima Morais DA, Chang L, Barrette M, et al. MetaboAnalyst 5.0: narrowing the gap between raw spectra and functional insights. Nucleic Acids Res. 2021;49(W1):W388–W396. doi: 10.1093/nar/gkab382 34019663 PMC8265181

[37] Thawornkuno C, Nogrado K, Adisakwattana P, Thiangtrongjit T, Reamtong O. Identification and profiling of Trichinella spiralis circulating antigens and proteins in sera of mice with trichinellosis. PLoS One. 2022;17(3):e0265013. doi: 10.1371/journal.pone.0265013 35271623 PMC8912135

[38] Kanehisa M, Furumichi M, Tanabe M, Sato Y, Morishima K. KEGG: new perspectives on genomes, pathways, diseases and drugs. Nucleic Acids Res. 2017;45(D1):D353–D361. doi: 10.1093/nar/gkw1092 27899662 PMC5210567

[39] Wishart DS, Guo A, Oler E, Wang F, Anjum A, Peters H, et al. HMDB 5.0: the Human Metabolome Database for 2022. Nucleic Acids Res. 2022;50(D1):D622–D631. doi: 10.1093/nar/gkab1062 34986597 PMC8728138

[40] Hastings J, Owen G, Dekker A, Ennis M, Kale N, Muthukrishnan V, et al. ChEBI in 2016: Improved services and an expanding collection of metabolites. Nucleic Acids Res. 2016;44(D1):D1214–D1219. doi: 10.1093/nar/gkv1031 26467479 PMC4702775

[41] Haug K, Cochrane K, Nainala VC, Williams M, Chang J, Jayaseelan KV, et al. MetaboLights: a resource evolving in response to the needs of its scientific community. Nucleic Acids Res. 2020;48(D1):D440–D444. doi: 10.1093/nar/gkz1019 31691833 PMC7145518

[42] Mitreva M, Jasmer DP. Biology and genome of Trichinella spiralis. WormBook. 2006;1–21. doi: 10.1895/wormbook.1.124.1 18050431 PMC4781409

[43] Bruschi F, Chiumiento L. Trichinella inflammatory myopathy: host or parasite strategy?. Parasit Vectors. 2011;4:42. doi: 10.1186/1756-3305-4-42 21429196 PMC3079684

[44] Battelli MG, Polito L, Bortolotti M, Bolognesi A. Xanthine Oxidoreductase-Derived Reactive Species: Physiological and Pathological Effects. Oxid Med Cell Longev. 2016;2016:3527579. doi: 10.1155/2016/3527579 26823950 PMC4707389

[45] Cui L, Fang J, Ooi EE, Lee YH. Serial Metabolome Changes in a Prospective Cohort of Subjects with Influenza Viral Infection and Comparison with Dengue Fever. J Proteome Res. 2017;16(7):2614–2622. doi: 10.1021/acs.jproteome.7b00173 28560878

[46] Bennuru S, Lustigman S, Abraham D, Nutman TB. Metabolite profiling of infection-associated metabolic markers of onchocerciasis. Mol Biochem Parasitol. 2017;215:58–69. doi: 10.1016/j.molbiopara.2017.01.008 28188804 PMC5474354

[47] Chienwichai P, Nogrado NS, Tipthara P, Tarning J, Limpanont Y, Chusongsang P, et al. Untargeted serum metabolomic profiling for early detection of *Schistosoma mekongi* infection in mouse model. Front Cell Infect Microbiol. 2022;1201. doi: 10.3389/fcimb.2022.910177 36061860 PMC9433908

[48] Xiong YJ, Chu HW, Lin Y, Han F, Li YC, Wang AG, et al. Hesperidin alleviates rat postoperative ileus through anti-inflammation and stimulation of Ca(2+)-dependent myosin phosphorylation. Acta Pharmacol Sin. 2016;37(8):1091–1100. doi: 10.1038/aps.2016.56 27345626 PMC4973386

[49] Biesemann N, Ried JS, Ding-Pfennigdorff D, Dietrich A, Rudolph C, Hahn S, et al. High throughput screening of mitochondrial bioenergetics in human differentiated myotubes identifies novel enhancers of muscle performance in aged mice. Sci Rep. 2018;8(1):9408. doi: 10.1038/s41598-018-27614-8 29925868 PMC6010423

[50] Tomšíková H, Tomšík P, Solich P, Nováková L. Determination of pteridines in biological samples with an emphasis on their stability. Bioanalysis. 2013;5(18):2307–2326. doi: 10.4155/bio.13.194 24053245

[51] Bonnet J, Vignoles P, Tiberti N, Gedeão V, Hainard A, Turck N, et al. Neopterin and CXCL-13 in Diagnosis and Follow-Up of Trypanosoma brucei gambiense Sleeping Sickness: Lessons from the Field in Angola. Biomed Res Int. 2019;2019:6070176. doi: 10.1155/2019/6070176 31886231 PMC6914994

[52] Kip AE, Wasunna M, Alves F, Schellens JHM, Beijnen JH, Musa AM, et al. Macrophage Activation Marker Neopterin: A Candidate Biomarker for Treatment Response and Relapse in Visceral Leishmaniasis. Front Cell Infect Microbiol. 2018;8:181. doi: 10.3389/fcimb.2018.00181 29911074 PMC5992270

[53] Li YX, Lu YP, Tang D, Hu B, Zhang ZY, Wu HW, et al. Anthocyanin improves kidney function in diabetic kidney disease by regulating amino acid metabolism. J Transl Med. 2022;20(1):510. doi: 10.1186/s12967-022-03717-9 36335368 PMC9636632

[54] Spick M, Campbell A, Baricevic-Jones I, von Gerichten J, Lewis HM, Frampas CF, et al. Multi-Omics Reveals Mechanisms of Partial Modulation of COVID-19 Dysregulation by Glucocorticoid Treatment. Int J Mol Sci. 2022;23(20):12079. doi: 10.3390/ijms232012079 36292938 PMC9602480

[55] Trostchansky A, Wood I, Rubbo H. Regulation of arachidonic acid oxidation and metabolism by lipid electrophiles. Prostaglandins Other Lipid Mediat. 2021;152:106482. doi: 10.1016/j.prostaglandins.2020.106482 33007446

[56] Kasatkina LA, Rittchen S, Sturm EM. Neuroprotective and Immunomodulatory Action of the Endocannabinoid System under Neuroinflammation. Int J Mol Sci. 2021;22(11):5431. doi: 10.3390/ijms22115431 34063947 PMC8196612

[57] Yang Y, Liu L, Liu X, Zhang Y, Shi H, Jia W, et al. Extracellular Vesicles Derived From *Trichinella spiralis* Muscle Larvae Ameliorate TNBS-Induced Colitis in Mice. Front Immunol. 2020;11:1174. doi: 10.3389/fimmu.2020.01174 32595641 PMC7300183

[58] Gao X, Yang Y, Liu X, Wang Y, Yang Y, Boireau P, et al. Extracellular vesicles derived from Trichinella spiralis prevent colitis by inhibiting M1 macrophage polarization. Acta Trop. 2021;213:105761. doi: 10.1016/j.actatropica.2020.105761 33221281

[59] Alzahrani FA, Shait Mohammed MR, Alkarim S, Azhar EI, El-Magd MA, Hawsawi Y, et al. Untargeted Metabolic Profiling of Extracellular Vesicles of SARS-CoV-2-Infected Patients Shows Presence of Potent Anti-Inflammatory Metabolites. Int J Mol Sci. 2021;22(19):10467. doi: 10.3390/ijms221910467 34638812 PMC8509011

[60] Cornford EM, Fitzpatrick AM. Comparative glucose utilization rates in separated and mated schistosomes. Exp Parasitol. 1987;64(3):448–457. doi: 10.1016/0014-4894(87)90059-2 3678450

[61] Valentine WJ, Hashidate-Yoshida T, Yamamoto S, Shindou H. Biosynthetic Enzymes of Membrane Glycerophospholipid Diversity as Therapeutic Targets for Drug Development. Adv Exp Med Biol. 2020;1274:5–27. doi: 10.1007/978-3-030-50621-6_2 32894505

[62] Rubio-Tomás T, Tavernarakis N. Lipid metabolism and ageing in Caenorhabditis elegans: a complex interplay. Biogerontology. 2022;23(5):541–557. doi: 10.1007/s10522-022-09989-4 36048312

[63] Harder A. The Biochemistry of Haemonchus contortus and Other Parasitic Nematodes. Adv Parasitol. 2016;93:69–94. doi: 10.1016/bs.apar.2016.02.010 27238003

[64] Mangmee S, Adisakwattana P, Tipthara P, Simanon N, Sonthayanon P, Reamtong O. Lipid profile of Trichinella papuae muscle-stage larvae. Sci Rep. 2020;10(1):10125. doi: 10.1038/s41598-020-67297-8 32576934 PMC7311410

[65] Liu R, Ye F, Zhong QP, Wang SH, Chai T, Dong HF, et al. Comparative serum metabolomics between SCID mice and BALB/c mice with or without Schistosoma japonicum infection: Clues to the abnormal growth and development of schistosome in SCID mice. Acta Trop. 2019;200:105186. doi: 10.1016/j.actatropica.2019.105186 31542371

[66] Huang Y, Wu Q, Zhao L, Xiong C, Xu Y, Dong X, et al. UHPLC-MS-Based Metabolomics Analysis Reveals the Process of Schistosomiasis in Mice. Front Microbiol. 2020;11:1517. doi: 10.3389/fmicb.2020.01517 32760365 PMC7371968

[67] Globisch D, Moreno AY, Hixon MS, Nunes AA, Denery JR, Specht S, et al. Onchocerca volvulus-neurotransmitter tyramine is a biomarker for river blindness. Proc Natl Acad Sci U S A. 2013;110(11):4218–4223. doi: 10.1073/pnas.1221969110 23440222 PMC3600455

[68] Globisch D, Eubanks LM, Shirey RJ, Pfarr KM, Wanji S, Debrah AY, et al. Validation of onchocerciasis biomarker N-acetyltyramine-O-glucuronide (NATOG). Bioorg Med Chem Lett. 2017;27(15):3436–3440. doi: 10.1016/j.bmcl.2017.05.082 28600214 PMC5510726

[69] Shirey RJ, Globisch D, Eubanks LM, Hixon MS, Janda KD. Noninvasive Urine Biomarker Lateral Flow Immunoassay for Monitoring Active Onchocerciasis. ACS Infect Dis. 2018;4(10):1423–1431. doi: 10.1021/acsinfecdis.8b00163 30141624 PMC6189908

[70] Jiang N, Zhang G, Pan L, Yan C, Zhang L, Weng Y, et al. Potential plasma lipid biomarkers in early-stage breast cancer. Biotechnol Lett. 2017;39(11):1657–1666. doi: 10.1007/s10529-017-2417-z 28828718

[71] Martín-Blázquez A, Díaz C, González-Flores E, Franco-Rivas D, Jiménez-Luna C, Melguizo C, et al. Untargeted LC-HRMS-based metabolomics to identify novel biomarkers of metastatic colorectal cancer. Sci Rep. 2019;9(1):20198. doi: 10.1038/s41598-019-55952-8 31882610 PMC6934557

[72] Chen S, Wu Q, Zhu L, Zong G, Li H, Zheng H, et al. Plasma glycerophospholipid profile, erythrocyte n-3 PUFAs, and metabolic syndrome incidence: a prospective study in Chinese men and women [published correction appears in Am J Clin Nutr. 2021 Jul 1;114(1):397]. Am J Clin Nutr. 2021;114(1):143–153. doi: 10.1093/ajcn/nqab050 33829226

[73] Wang S, Tang K, Lu Y, Tian Z, Huang Z, Wang M, et al. Revealing the role of glycerophospholipid metabolism in asthma through plasma lipidomics. Clin Chim Acta. 2021;513:34–42. doi: 10.1016/j.cca.2020.11.026 33307061

[74] Wewer V, Makepeace BL, Tanya VN, Peisker H, Pfarr K, Hoerauf A, et al. Lipid profiling of the filarial nematodes Onchocerca volvulus, Onchocerca ochengi and Litomosoides sigmodontis reveals the accumulation of nematode-specific ether phospholipids in the host. Int J Parasitol. 2017;47(14):903–912. doi: 10.1016/j.ijpara.2017.06.001 28743489 PMC5716430

[75] Adebayo AS, Mundhe SD, Awobode HO, Onile OS, Agunloye AM, Isokpehi RD, et al. Metabolite profiling for biomarkers in Schistosoma haematobium infection and associated bladder pathologies. PLoS Negl Trop Dis. 2018;12(4):e0006452. doi: 10.1371/journal.pntd.0006452 29708967 PMC5945272

[76] Retra K, deWalick S, Schmitz M, Yazdanbakhsh M, Tielens AG, Brouwers JF, et al. The tegumental surface membranes of Schistosoma mansoni are enriched in parasite-specific phospholipid species. Int J Parasitol. 2015;45(9–10):629–636. doi: 10.1016/j.ijpara.2015.03.011 25975668

