# Supplementary figures and images for: Untargeted serum metabolomics analysis of Trichinella spiralis-infected mouse
Source: PLoS Negl Trop Dis. 2023 Feb 21;17(2):e0011119. doi: 10.1371/journal.pntd.0011119 (PMC9943014; doi:10.1371/journal.pntd.0011119)

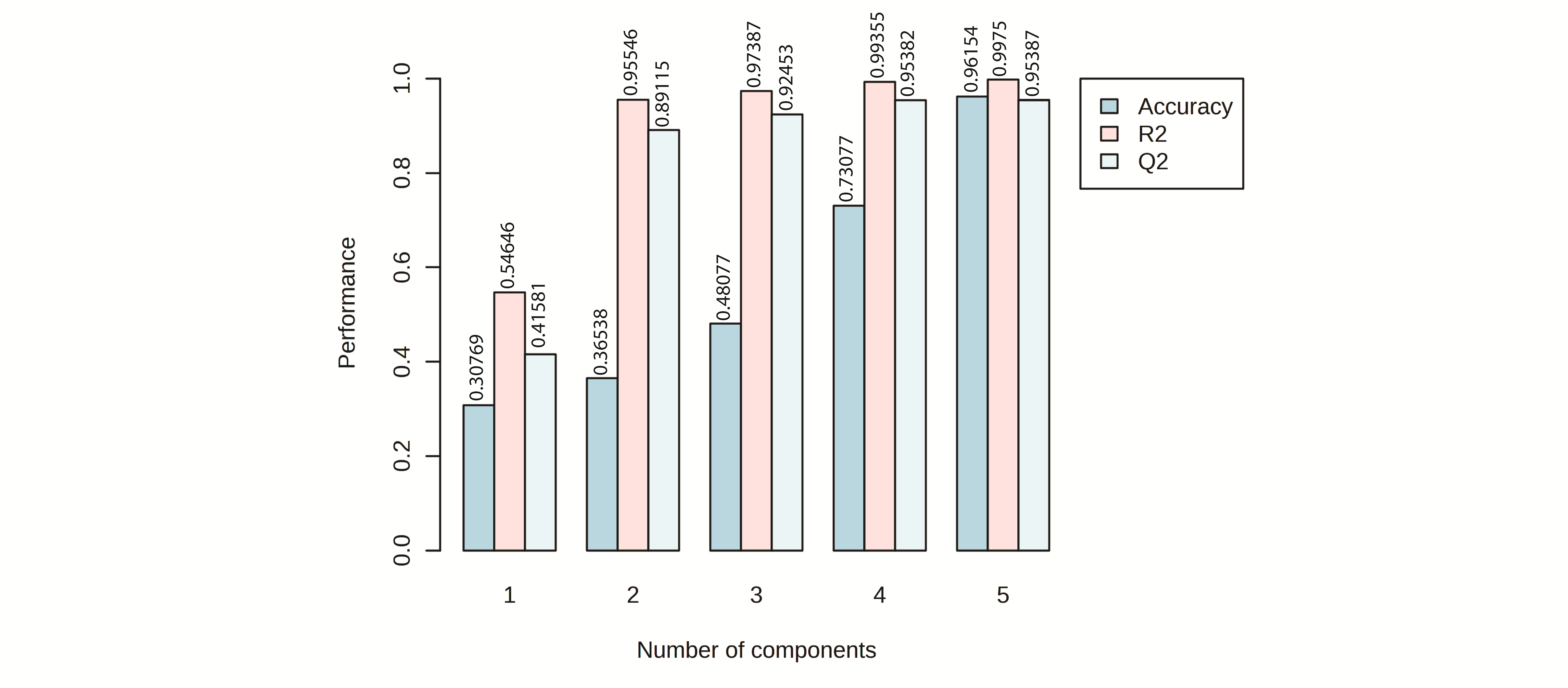

Supplement: S2 Fig — The validation method shows cumulative values of R2 = 0.9975 and Q2 = 0.95387 for 5 components. This cross validation indicates good prediction and less likely for model overfitting. (TIF) [file pntd.0011119.s002.tif]

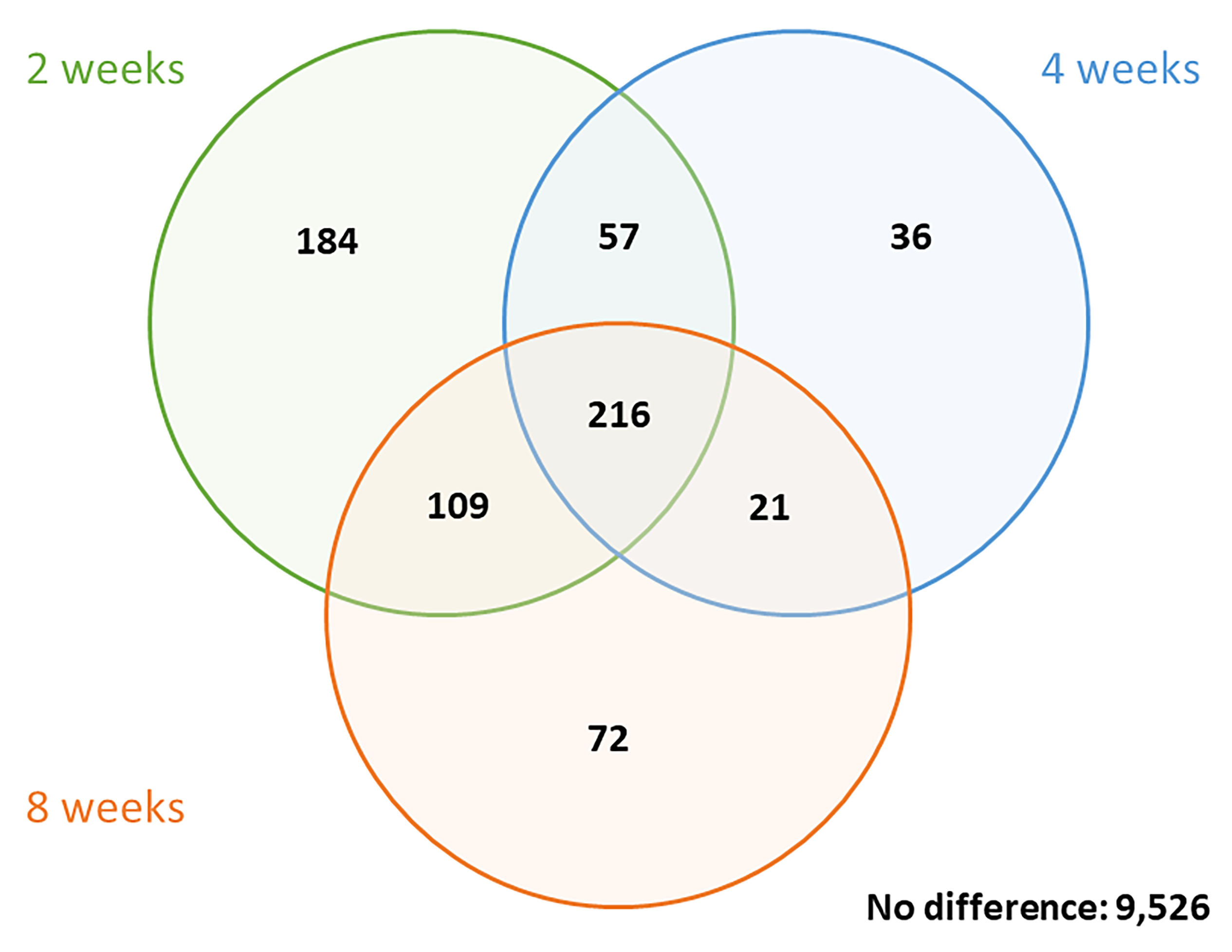

Supplement: S3 Fig — Green, blue, and orange circles represent differential metabolites at 2-, 4-, and 8-weeks PI, respectively. (TIF) [file pntd.0011119.s003.tif]

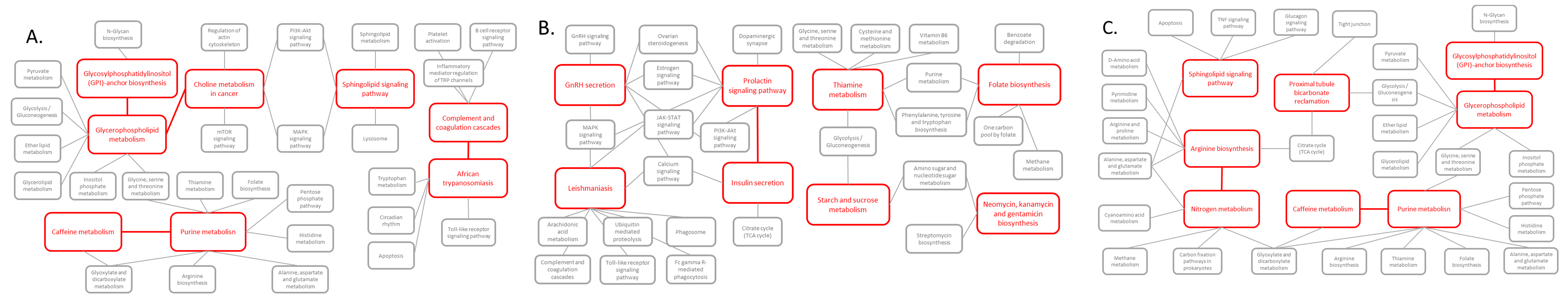

Supplement: S4 Fig — (A) Interconnection generated from integrated pathway analysis of data from 2-weeks PI. (B) Interconnection generated from integrated pathway analysis of data from 4-weeks PI. (C) Interconnection generated from integrated pathway analysis of data from 8-weeks PI. Red nodes represent identified pathways. Gray nodes represent related pathways. Only pathways with interconnections are presented. Many identified pathways are linked directly or indirectly via the same related pathways, indicating profound effects of infection to host body. (TIF) [file pntd.0011119.s004.tif]
