# Supplementary material for: Untargeted serum metabolomics analysis of Trichinella spiralis-infected mouse
Source: PLoS Negl Trop Dis. 2023 Feb 21;17(2):e0011119. doi: 10.1371/journal.pntd.0011119 (PMC9943014; doi:10.1371/journal.pntd.0011119)
Supplement: S1 Table — (DOCX) [file pntd.0011119.s005.docx]

**Supplementary Table S1.** Number of significantly changed metabolomic features in each time-point

|  | **Increased features** | | | **Decreased features** | | | **Total features** |
| --- | --- | --- | --- | --- | --- | --- | --- |
|  | + mode | - mode | Total | + mode | - mode | Total |  |
| **2 weeks PI** | 36 | 5 | 41 | 387 | 138 | 525 | 566 |
| **4 weeks PI** | 36 | 8 | 44 | 225 | 61 | 286 | 330 |
| **8 weeks PI** | 45 | 22 | 67 | 259 | 92 | 351 | 418 |
