# Supplementary material for: Untargeted serum metabolomics analysis of Trichinella spiralis-infected mouse
Source: PLoS Negl Trop Dis. 2023 Feb 21;17(2):e0011119. doi: 10.1371/journal.pntd.0011119 (PMC9943014; doi:10.1371/journal.pntd.0011119)
Supplement: S3 Table — (DOCX) [file pntd.0011119.s007.docx]

**Supplementary Table S4.** Percentage and enrichment *p-*value of significantly changed metabolites in each time-point

| Chemical Structure | 2 weeks PI | | 4 weeks PI | | 8 weeks PI | |
| --- | --- | --- | --- | --- | --- | --- |
|  | % | Enrichment *p*-value | % | Enrichment *p*-value | % | Enrichment *p*-value |
| Super-class | | | | | | |
| Lipids and lipid-like molecules | 28.0 | 0.993 | 21.7 | 0.994 | 36.0 | 0.935 |
| Glycerophospholipids | 24.0 | 0.238 | 13.0 | 0.679 | 20.0 | 0.386 |
| Fatty Acyls | 16.0 | 0.0365 | 21.7 | 0.00306 | 8.0 | 0.349 |
| Sterol Lipids | 8.0 | 0.0532 | 8.7 | 0.036 | 12.0 | 0.00551 |
| Carbohydrates | 4.0 | 0.021 | 13.0 | 7.15E-07 | 4.0 | 0.0203 |
| Organic acids | 4.0 | 0.133 | 8.7 | 0.00592 | 0.0 | - |
| Organoheterocyclic compounds | 4.0 | 0.0707 | 4.3 | 0.0574 | 0.0 | - |
| Polyketides | 4.0 | 0.584 | 4.3 | 0.507 | 4.0 | 0.572 |
| Sphingolipids | 4.0 | 0.99 | 0.0 | - | 4.0 | 0.988 |
| Nucleic acids | 4.0 | 0.028 | 0.0 | - | 4.0 | 0.0271 |
| Alkaloids | 0.0 | - | 4.3 | 0.00653 | 4.0 | 0.00783 |
| Prenol Lipids | 0.0 | - | 0.0 | - | 4.0 | 0.207 |
| Main-class | | | | | | |
| Glycerophospholipids | 15.4 | 0.846 | 4.3 | 0.994 | 15.4 | 0.827 |
| Glycerophosphoserines | 19.2 | 0.000427 | 13.0 | 0.0148 | 11.5 | 0.0242 |
| Glycerophosphoethanolamines | 0.0 | - | 0.0 | - | 3.8 | 0.486 |
| Glycerophosphocholines | 3.8 | 0.525 | 0.0 | - | 3.8 | 0.513 |
| Glycerolipids | 0.0 | - | 0.0 | - | 3.8 | 0.999 |
| Phosphosphingolipids | 3.8 | 0.532 | 0.0 | - | 3.8 | 0.52 |
| Sphingomyelins | 3.8 | 0.305 | 0.0 | - | 3.8 | 0.297 |
| Fatty Acyls | 7.7 | 0.0222 | 13.0 | 0.000832 | 7.7 | 0.0209 |
| Fatty Acids and Conjugates | 7.7 | 0.0836 | 4.3 | 0.324 | 3.8 | 0.375 |
| Fatty esters | 3.8 | 0.255 | 13.0 | 0.00165 | 0.0 | - |
| Octadecanoids | 3.8 | 0.0749 | 4.3 | 0.0609 | 0.0 | - |
| Prenol lipids | 0.0 | - | 0.0 | - | 3.8 | 0.313 |
| Sterols | 7.7 | 0.00771 | 8.7 | 0.00506 | 7.7 | 0.00723 |
| Steroids | 3.8 | 0.151 | 4.3 | 0.124 | 3.8 | 0.147 |
| Flavonoids | 3.8 | 0.568 | 4.3 | 0.492 | 3.8 | 0.556 |
| Eicosanoids | 0.0 | - | 0.0 | - | 3.8 | 0.14 |
| Isoprenoids | 0.0 | - | 0.0 | - | 3.8 | 0.194 |
| Benzophenanthridine alkaloids | 0.0 | - | 4.3 | 0.000378 | 3.8 | 0.000453 |
| Monosaccharides | 3.8 | 0.015 | 13.0 | 2.58E-07 | 3.8 | 0.0146 |
| Purines | 3.8 | 0.0138 | 0.0 | - | 3.8 | 0.0134 |
| Indoles | 3.8 | 0.00561 | 4.3 | 0.00453 | 0.0 | - |
| Amino acids and peptides | 0.0 | - | 4.3 | 0.0872 | 0.0 | - |
| Bile acids | 0.0 | - | 0.0 | - | 3.8 | 0.046 |
| Organic dicarboxylic acids | 3.8 | 0.00359 | 4.3 | 0.00289 | 0.0 | - |
| Sub-class | | | | | | |
| Diacylglycerophosphoserines | 17.9 | 2.84E-07 | 12.0 | 0.000195 | 11.5 | 0.000339 |
| Glycerophosphocholines | 7.1 | 0.00265 | 0.0 | - | 7.7 | 0.00248 |
| Glycerophosphoserines | 3.6 | 0.0646 | 4.0 | 0.0525 | 3.8 | 0.0626 |
| Glycerophosphoethanolamines | 3.6 | 0.317 | 0.0 | - | 3.8 | 0.308 |
| Diacylglycerophosphoethanolamines | 0.0 | - | 0.0 | - | 3.8 | 0.137 |
| Diacylglycerophosphocholines | 0.0 | - | 0.0 | - | 3.8 | 0.178 |
| Ether Lysophosphatidylcholines | 3.6 | 0.00265 | 0.0 | - | 0.0 | - |
| Ceramide phosphocholines | 3.6 | 0.00125 | 0.0 | - | 3.8 | 0.00121 |
| Unsaturated Fatty Acids | 0.0 | - | 0.0 | - | 3.8 | 0.0396 |
| Hydroxy Fatty Acids | 0.0 | - | 4.0 | 0.034 | 0.0 | - |
| Hydroxyeicosatetraenoic acid | 0.0 | - | 0.0 | - | 3.8 | 0.00904 |
| Fatty alcohols | 7.1 | 0.00231 | 12.0 | 2.61E-05 | 7.7 | 0.00216 |
| Fatty acyl carnitines | 3.6 | 0.0141 | 8.0 | 6.21E-05 | 0.0 | - |
| Amino Fatty Acids | 3.6 | 0.00359 | 0.0 | - | 0.0 | - |
| Hydroxyoctadecadienoic acids | 3.6 | 0.00281 | 4.0 | 0.00227 | 0.0 | - |
| Acyl carnitines | 0.0 | - | 8.0 | 0.000253 | 0.0 | - |
| Acylaminosugars | 0.0 | - | 4.0 | 0.000756 | 0.0 | - |
| Diradylglycerols | 0.0 | - | 0.0 | - | 3.8 | 0.311 |
| Oxysterols | 7.1 | 1.75E-05 | 8.0 | 1.13E-05 | 7.7 | 1.63E-05 |
| Sphingomyelins | 3.6 | 0.305 | 0.0 | - | 3.8 | 0.297 |
| Cholestane steroids | 3.6 | 0.00499 | 4.0 | 0.00403 | 3.8 | 0.00483 |
| Triterpenoids | 0.0 | - | 0.0 | - | 3.8 | 0.0745 |
| C15 isoprenoids | 0.0 | - | 0.0 | - | 3.8 | 0.0492 |
| Other Octadecanoids | 3.6 | 0.0402 | 4.0 | 0.0325 | 0.0 | - |
| Flavonoid glycosides | 3.6 | 0.00328 | 4.0 | 0.00264 | 3.8 | 0.00317 |
| Flavanones | 3.6 | 0.0789 | 4.0 | 0.0641 | 3.8 | 0.0765 |
| Xanthines | 3.6 | 0.00281 | 0.0 | - | 3.8 | 0.00272 |
| Indoles | 3.6 | 0.00234 | 4.0 | 0.00189 | 0.0 | - |
| Sugar acids | 3.6 | 0.00359 | 4.0 | 0.0029 | 3.8 | 0.00347 |
| Monosaccharide phosphates | 0.0 | - | 4.0 | 0.00302 | 0.0 | - |
| Amino acids | 0.0 | - | 4.0 | 0.0343 | 0.0 | - |
| C24 bile acids | 0.0 | - | 0.0 | - | 3.8 | 0.0224 |
| Dicarboxylic acids | 3.6 | 0.0217 | 0.0 | - | 0.0 | - |
| Organic dicarboxylic acids | 3.6 | 0.00359 | 4.0 | 0.0029 | 0.0 | - |
