# Supplementary material for: Untargeted serum metabolomics analysis of Trichinella spiralis-infected mouse
Source: PLoS Negl Trop Dis. 2023 Feb 21;17(2):e0011119. doi: 10.1371/journal.pntd.0011119 (PMC9943014; doi:10.1371/journal.pntd.0011119)
Supplement: S6 Table — (DOCX) [file pntd.0011119.s010.docx]

**Supplementary Table S6. Potential glycerophospholipid markers those were not identified from human and mouse databases.**

| **No** | **Glycerophospholipid species** | **METLIN ID** | **AUC Value** | | | ***p-*value** | | | **Fold-change** | | |
| --- | --- | --- | --- | --- | --- | --- | --- | --- | --- | --- | --- |
|  |  |  | **2-week PI** | **4-week PI** | **8-week PI** | **2-week PI** | **4-week PI** | **8-week PI** | **2-week PI** | **4-week PI** | **8-week PI** |
| 1 | PE(19:0/16:0) | 40568 | 1.00 | 1.00 | 1.00 | 7.20E^-08^ | 2.48E^-07^ | 1.27E^-06^ | 3.40 | 2.89 | 2.65 |
| 2 | PS(18:1(9Z)/18:1(9Z))[U] | 40798 | 1.00 | 1.00 | 1.00 | 2.84E^-06^ | 1.81E^-04^ | 1.04E^-08^ | -3.20 | -2.23 | -3.31 |
| 3 | PS(12:0/15:0) | 77711 | 1.00 | 1.00 | 1.00 | 7.18E^-06^ | 4.93E^-05^ | 6.81E^-08^ | -4.69 | -3.33 | -4.58 |
| 4 | PG(O-18:0/22:6(4Z,7Z,10Z,13Z,16Z,19Z)) | 79894 | 1.00 | 1.00 | 1.00 | 6.43E^-06^ | 1.81E^-04^ | 4.21E^-08^ | -3.27 | -2.22 | -3.50 |
| 5 | PS(O-18:0/18:3(6Z,9Z,12Z)) | 78676 | 0.99 | 0.96 | 1.00 | 8.91E^-05^ | 0.0013 | 8.08E^-07^ | -3.63 | -2.29 | -3.58 |
| 6 | PAz-PC | 63020 | 0.98 | 0.96 | 1.00 | 1.43E^-05^ | 1.37E^-04^ | 9.81E^-08^ | -4.64 | -3.12 | -4.87 |
| 7 | PS(13:0/16:0) | 77736 | 0.99 | 0.95 | 1.00 | 1.02E^-05^ | 4.00E^-04^ | 1.20E^-06^ | -3.66 | -2.52 | -2.96 |
| 8 | PS(18:1(9Z)/18:2(9Z,12Z))[U] | 40803 | 0.98 | 0.94 | 1.00 | 1.37E^-04^ | 5.58E^-04^ | 7.64E^-08^ | -3.00 | -2.16 | -3.26 |
| 9 | PS(O-16:0/20:3(8Z,11Z,14Z)) | 78722 | 0.93 | 0.93 | 1.00 | 0.0012 | 0.0037 | 6.36E^-06^ | -2.93 | -2.07 | -3.14 |
| 10 | PE-Cer(d14:1(4E)/22:1(13Z)(2OH)) | 103102 | 0.84 | 0.93 | 1.00 | 0.0056 | 7.58E^-04^ | 1.20 E^-04^ | -2.93 | -3.04 | -3.26 |
| 11 | PS(18:2(9Z,12Z)/18:0)[U] | 40817 | 1.00 | 0.91 | 1.00 | 6.69E^-06^ | 0.0014 | 4.21E^-08^ | -3.09 | -2.11 | -3.18 |
| 12 | PS(16:0/22:2(13Z,16Z)) | 77864 | 0.94 | 0.91 | 1.00 | 8.23E^-05^ | 0.0015 | 4.49E^-08^ | -3.80 | -2.55 | -4.61 |
| 13 | PS(18:0/18:1(9Z))[U] | 40797 | 0.94 | 0.86 | 1.00 | 3.92E^-04^ | 0.0063 | 5.54E^-06^ | -2.77 | -2.01 | -2.45 |
| 14 | PC(O-10:0/O-10:0)[U] | 40181 | 0.95 | 0.99 | 0.99 | 1.15E^-04^ | 8.30E^-06^ | 1.42E^-05^ | -2.32 | -2.57 | -2.14 |
| 15 | PS(O-18:0/18:3(9Z,12Z,15Z)) | 78677 | 0.96 | 0.86 | 0.99 | 1.20E^-04^ | 0.0044 | 4.05E^-06^ | -3.60 | -2.32 | -3.61 |
| 16 | PS(O-16:0/20:1(11Z)) | 78659 | 0.80 | 0.86 | 0.99 | 0.0137 | 0.0027 | 2.20E^-04^ | -2.86 | -2.91 | -3.46 |
| 17 | PS(O-16:0/20:3(8Z,11Z,14Z)) | 78722 | 0.95 | 0.82 | 0.99 | 1.44E^-04^ | 0.0093 | 9.26E^-06^ | -3.83 | -2.17 | -3.52 |
| 18 | PE-Cer(d14:2(4E,6E)/24:1(15Z)) | 103076 | 0.96 | 0.96 | 0.96 | 5.61E^-04^ | 1.58E^-04^ | 8.56E^-04^ | 6.01 | 3.99 | 3.55 |
| 19 | PS(12:0/17:0) | 77714 | 0.90 | 0.91 | 0.96 | 0.0011 | 0.0012 | 6.56E^-05^ | -3.23 | -2.65 | -3.10 |
| 20 | PS(18:0/19:0)[U] | 40822 | 0.98 | 1.00 | 0.95 | 5.06E^-04^ | 2.17E^-04^ | 1.87E^-04^ | 2.40 | 2.88 | 2.09 |
| 21 | PS(13:0/17:1(9Z)) | 77738 | 0.95 | 0.89 | 0.95 | 6.62E^-05^ | 0.0035 | 3.55E^-04^ | -4.39 | -2.51 | -2.67 |
| 22 | PS(16:0/22:1(11Z)) | 77863 | 0.95 | 0.75 | 0.95 | 2.02E^-04^ | 0.0151 | 3.14E^-04^ | -3.44 | -2.12 | -2.77 |
| 23 | PS(18:1(9Z)/18:0)[U] | 40804 | 0.94 | 0.93 | 0.94 | 9.75E^-05^ | 5.94E^-04^ | 5.41E^-05^ | -4.44 | -3.12 | -3.60 |
| 24 | PA(14:0/14:0)[U] | 40908 | 0.95 | 0.91 | 0.94 | 2.89E^-04^ | 0.0016 | 6.40E^-04^ | -3.05 | -2.53 | -2.38 |
| 25 | PG(O-16:0/19:1(9Z)) | 79822 | 0.95 | 0.86 | 0.94 | 5.46E^-04^ | 0.0051 | 1.75E^-04^ | -3.35 | -2.22 | -2.93 |
| 26 | PS(O-16:0/20:2(11Z,14Z)) | 78660 | 0.83 | 0.89 | 0.93 | 0.0038 | 0.0035 | 1.49E^-04^ | -3.24 | -2.43 | -3.04 |
| 27 | PG(O-20:0/14:1(9Z)) | 79855 | 0.91 | 0.98 | 0.91 | 0.0070 | 0.0027 | 0.0184 | -3.02 | -2.94 | -2.24 |
| 28 | PS(12:0/20:2(11Z,14Z)) | 77724 | 0.91 | 0.86 | 0.91 | 3.86E^-04^ | 0.0058 | 4.80E^-04^ | -3.52 | -2.11 | -2.12 |
| 29 | PA(O-18:0/17:0) | 82171 | 0.93 | 0.93 | 0.89 | 9.80E^-04^ | 8.36E^-04^ | 0.0040 | -2.92 | -2.45 | -2.02 |
| 30 | PS(14:0/12:0) | 78595 | 0.83 | 0.86 | 0.89 | 0.0494 | 0.0310 | 0.0051 | -3.10 | -2.12 | -2.59 |
| 31 | PE-Cer(d14:1(4E)/22:0(2OH)) | 103101 | 0.86 | 0.93 | 0.84 | 0.0014 | 0.0028 | 0.0193 | 2.38 | 2.98 | 2.71 |
| 32 | PA(O-16:0/21:0) | 82161 | 0.80 | 0.83 | 0.83 | 0.0225 | 0.0135 | 0.0014 | 2.96 | 2.34 | 3.18 |
| 33 | PS(P-18:0/18:2(9Z,12Z)) | 78780 | 0.89 | 0.80 | 0.82 | 0.0066 | 0.0118 | 0.0154 | -2.70 | -2.02 | -2.07 |
| 34 | PG(19:0/22:2(13Z,16Z)) | 79302 | 0.83 | 0.79 | 0.73 | 0.0267 | 0.0209 | 0.0446 | 2.28 | 2.08 | 2.78 |
